# The Glymphatic–Meningeal Lymphatic System and Autophagy in ME/CFS and Long COVID: A Mechanistic Perspective

**DOI:** 10.3390/ijms27209351

**Published:** 2026-10-21

**Authors:** Mohsen Nemat-Gorgani, Michael Anthony Jensen, Ronald Wayne Davis

**Affiliations:** Stanford Genome Technology Center, Department of Biochemistry, Stanford University, Palo Alto, CA 94304, USA; m.a.jensen@stanford.edu (M.A.J.); ron.davis@standord.edu (R.W.D.)

**Keywords:** ME/CFS, Long COVID, fibromyalgia, pain, neuroimmune, neurological disorders, misfolded proteins, glymphatic/meningeal lymphatic system, autophagy, proteostasis

## Abstract

Myalgic encephalomyelitis/chronic fatigue syndrome (ME/CFS) and Post-Acute Sequelae of SARS-CoV-2 infection (Long COVID) are complex multisystem disorders affecting 60–400 million individuals globally. They are considered post-infectious illnesses and share similar biological mechanisms, including immune dysregulation, orthostatic intolerance and cognitive dysfunction, highlighting a substantial overlap between the two conditions. Persistent fatigue, sleep dysfunction, post-exertional malaise and brain fog are among some of their most common and debilitating symptoms. Despite extensive research, they remain incurable, with no effective treatments, or simple diagnostic tests. They are increasingly understood as heterogeneous, multisystem diseases, involving neurological symptoms, with varying severities and diverse underlying biological abnormalities. More recently, the possibility of glymphatic dysfunction and autophagy impairment have been proposed as parts of the mechanistic features of the pathophysiology of these conditions, which are discussed more fully in the present perspective. With the glymphatic and meningeal lymphatic systems being operationally integrated, we will try to delineate how the metabolic waste removal mechanism afforded by this system may work together with autophagy to maintain brain homeostasis. We will provide some of the established mechanistic features of toxicity of misfolded proteins, explore their role in causing glymphatic dysfunction and impairing autophagy, and discuss the potential for therapeutic interventions.

## 1. Introduction

ME/CFS and Long COVID share remarkably similar pathophysiology and symptom profiles, including neurological and cognitive symptoms. About 50% of Long COVID patients are generally believed to meet the diagnostic criteria for ME/CFS, with high degrees of overlap [1,2]. As an estimate, Long COVID and ME/CFS affect 60–400 million individuals, globally [3]. Some of the shared mechanisms include persistent neuroimmune dysregulation, chronic inflammation, mitochondrial and metabolic dysfunction and dysautonomia. Neurological and cognitive disturbances are reported in both conditions, including neuroinflammation [4] and reduced cerebral blood flow [5,6]. There is currently no known cure for ME/CFS or Long COVID, and finding effective treatments is difficult for these complex heterogeneous diseases. Accordingly, a greater mechanistic insight into these conditions may potentially lead to more effective treatments and the likelihood of finding a cure.

In the present perspective, we describe the potential significance of an intimate cooperation between the glymphatic–meningeal lymphatic system and the autophagy–lysosomal pathway for the removal of waste products in these two disorders toward maintaining tissue homeostasis, particularly within the central nervous system (CNS). This is in continuation of our interest related to the possibility of axonal damage (demyelination) occurring in ME/CFS [7], also found to occur in COVID-19 [8], and a report describing formation of prion-like amyloid aggregates from the degradation products of myelin basic protein [9]. More recently, we and others have pointed out the possible involvement of glymphatic dysregulation and its potential significance in ME/CFS [10,11] and Long COVID [10,11,12], which has previously been described to be a major contributory factor to neurological disorders; in recent studies involving the use of MRI-based methods, neuroimaging evidence suggests changes in glymphatic function among patients with ME/CFS [13] and Long COVID [14,15,16].

The finding that ME/CFS serum samples display a greater protein aggregation propensity [17], with amyloid fibrin microclots also occurring in ME/CFS and Long COVID [18,19,20], is of special significance related to the present perspective. These misfolded proteins are of beta-sheet-rich amyloid nature [21] with structural and behavioral features of prions [22]. Consequently, they may have the ability to induce misfolding of neighboring proteins, further highlighting the potential importance of an impaired autophagy in the pathophysiology of ME/CFS and Long COVID, already indicated by a number of studies. Misfolded protein aggregates in the form of amyloid (peptide) structures derived from EBV proteins [23] and SARS-CoV-2 spike protein [24] have also been reported. We present the argument that all these and similar findings should be closely analyzed for a better understanding of the role of protein misfolding in causing toxicity and make an attempt towards providing a mechanistic insight into the potential significance of impairment/dysfunction of the removal mechanisms.

Autophagy impairment has also been described to play a critical role in the pathophysiology of a diverse number of conditions, including neurodegenerative [25], infectious [26], and autoimmune [27] diseases. However, in most of these studies, some specific aspects of general autophagy (macroautophagy) have been described without delineating the role of specific types and the related mechanisms, which we believe are essential for a better understanding of the molecular basis of the events. Accordingly, we will make an attempt toward elucidating some of the functional aspects of the mechano-biochemical features of the glymphatic–meningeal lymphatic and autophagy waste removal system in these conditions and discuss how autophagy impairment may act as a critical bottleneck, by taking a mechanistic approach. In doing so, we will include a discussion on the potential sources of misfolded proteins and factors affecting their formation and propagation. Furthermore, we will analyze some of the proposed dysfunctional aspects of different types of autophagy in specific cell types, the consequences of misfolded protein accumulation in these cells, and their potential roles in the pathophysiology of ME/CFS and Long COVID. We will also point out the significance of these events related to the underlying biology of fibromyalgia, with which they have important overlap [28,29], and then we will conclude with suggested potential therapeutic interventions.

This perspective extends our earlier proposal that glymphatic dysfunction contributes to ME/CFS [11]. We provide a detailed mechanistic insight of autophagy: rather than treating autophagy as one undifferentiated process, we distinguish among its specific subtypes (e.g., mitophagy, lysophagy, aggrephagy, ER-phagy) and examine their dysfunction across specific CNS and immune cell types, using misfolded protein aggregation as the common substrate with the ability to link glymphatic and autophagic waste clearance systems.

## 2. The Glymphatic–Meningeal Lymphatic System

**Figure 1:**
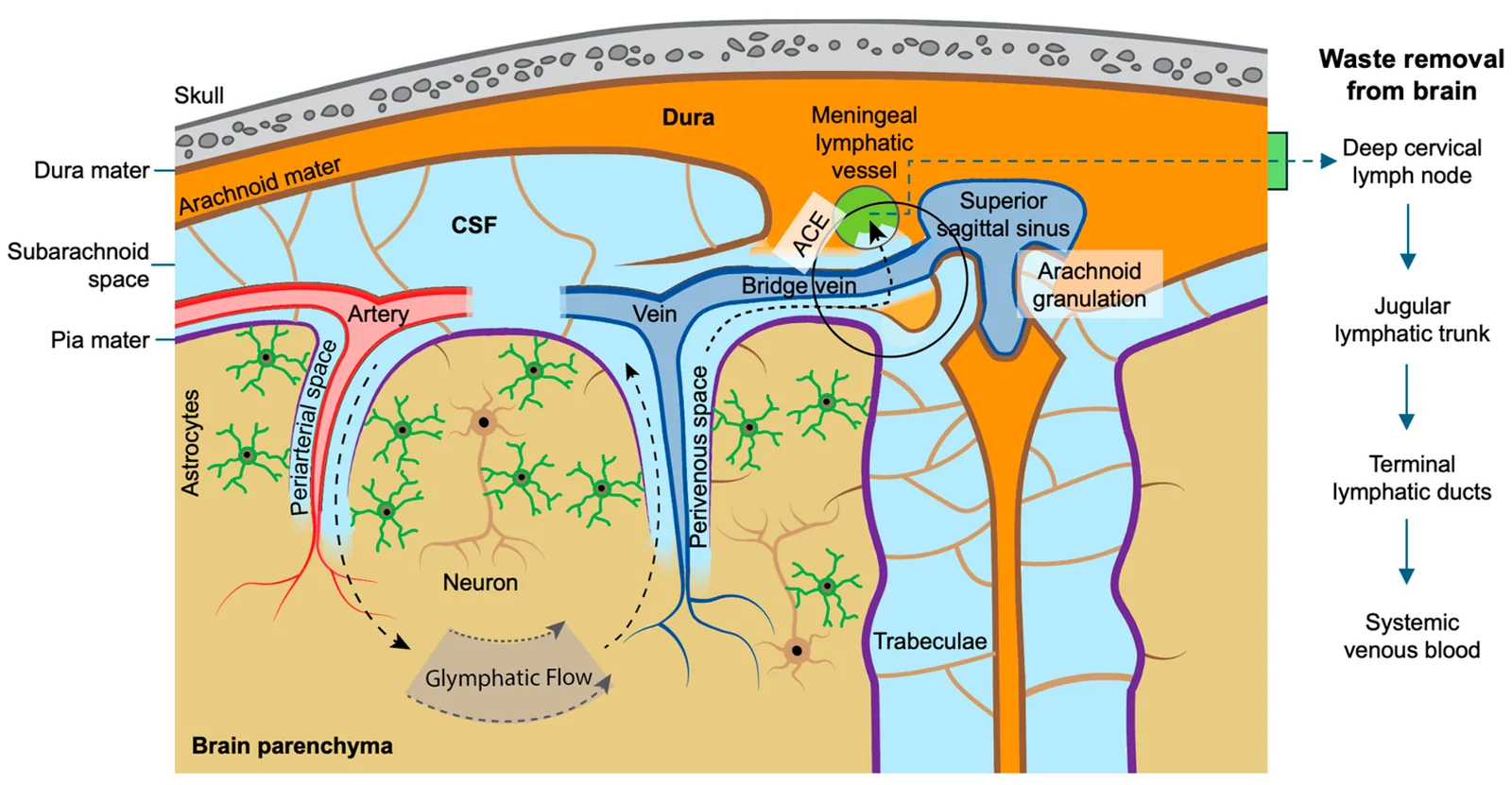
The anatomy of the brain parenchymal waste removal process. Neurons and astrocytes release metabolic waste into the brain interstitial fluid. CSF entering along periarterial spaces exchanges with this interstitial fluid (aided by astrocytic endfeet/aquaporin-4 water channels) [35] and carries solutes into perivenous spaces, returning them to the subarachnoid space—the glymphatic circuit shown by the dashed arrows. From the subarachnoid space, waste-laden fluid reaches venous blood by two parallel routes: directly, via arachnoid granulations emptying into the superior sagittal sinus [36,37,38], or via arachnoid cuff exits (ACE) surrounding bridging veins [39], which feed the dural meningeal lymphatic vessels [40,41]. Fluid draining via meningeal lymphatics reaches the deep cervical lymph nodes, then converges into the jugular lymphatic trunk, passes through the terminal lymphatic ducts (thoracic duct/right lymphatic duct), and empties into systemic venous blood at the jugulosubclavian junction. Both routes—direct arachnoid granulation absorption and the meningeal lymphatic/cervical node pathway—ultimately converge on the venous system as the final common destination for waste removed from the brain.

The glymphatic system and the meningeal lymphatic vessels act as a continuous collaborative axis for preserving brain health. This is achieved through the ability of the glymphatic system in “pumping” cerebrospinal fluid through the brain parenchyma and “pushing” the fluid waste into the subarachnoid space and the meningeal lymphatic vessels. The waste material is then transported out of the CNS into the deep cervical lymph nodes, thereby completing the mechanical part of this waste removal “pipeline” (Figure 1).

One of the important aspects of this waste removal mechanism is the “drainage” capacity of the meningeal lymphatics and the fact that the glymphatic and meningeal lymphatic systems are anatomically and functionally connected [30]. This interconnected and comprehensive “glymphatic–meningeal lymphatic clearance pathway” serves as a key communicator and a vital bridge between the brain and the body’s peripheral immune system [31,32,33]. Impairment in any of the components within this pathway believed to occur in neurological dysfunctions can result in accumulation of toxic waste products, such as misfolded protein aggregates, leading to cognitive decline and neuroinflammation [30,33,34].

Connected to the glymphatic–lymphatic system is the protein quality control mechanism or proteostasis, the cellular balance of protein production, folding and degradation. This connection is critical because it represents the brain’s waste removal pathway, preventing the accumulation of misfolded proteins that could otherwise cause pathology, like neurodegenerative diseases.

## 3. Proteostasis and Autophagy

Cellular protein homeostasis, or proteostasis, is a dynamic biological process that regulates the production, folding and degradation of proteins within a cell. It ensures the body’s proteome is kept functional by preventing the buildup of toxic, misfolded protein aggregates. Accordingly, it plays a pivotal role in preserving cellular health, and any disruption in this highly regulated process may lead to various protein misfolding diseases.

Cellular protein quality control is an intricate network that maintains proteostasis by folding, refolding, or destroying damaged proteins. It relies on three primary, interconnected pillars: molecular chaperones, the ubiquitin–proteasome system (UPS), and autophagy [42,43,44]. These three systems operate in a highly cooperative and synchronized manner to prevent the accumulation of toxic protein aggregates and the related pathologies, commonly described as proteinopathies [45]. UPS and autophagy are the two main proteolytic systems, sharing the commonality of utilizing ubiquitin tagging to target misfolded, damaged or superfluous proteins/organelles for degradation. While UPS degrades mostly smaller, soluble short-lived proteins, the autophagy–lysosomal pathway is responsible for clearing larger, insoluble long-lived aggregated proteins and organelles. When compromised, they fail to prevent protein aggregation, and may exacerbate the process, becoming actively detrimental rather than protective to the cells. Accordingly, these two highly regulated pathways not only maintain protein homeostasis, but also act as mediators and regulators of apoptosis and necrosis. Disruption of proteostasis is now recognized as a unifying pathological feature of neurodegenerative disorders, cancer, cardiovascular and metabolic diseases, and autoimmune conditions [46].

Autophagy is a highly conserved cellular “self-eating” catalytic process that safeguards proteostasis. It involves the formation of a double-membrane organelle called autophagosome, and functions as a vital intracellular degradation system for waste clearance in brain cells [47,48]. At only 2% of total body weight, the brain is a highly metabolic organ, consuming roughly 20% of the body’s total energy. This, together with the fact that neurons cannot divide, makes them heavily reliant on autophagy to clear damaged mitochondria and misfolded proteins for the prevention of neurological problems. Accordingly, any impairment in autophagy is potentially relevant to the neurological aspects of ME/CFS and Long COVID.

The term autophagy was coined by the Belgian biochemist Christian de Duve in 1963, who discovered that lysosomes have the capacity to destroy their own contents using lysosomal enzymes [49]. It was later established that autophagy participates in the breaking down and removal of waste material in the brain by degrading protein aggregates and damaged organelles [47,48]. Lysosomes contain about 50 hydrolytic enzymes, capable of breaking down proteins, lipids, polynucleotides and carbohydrates. The acidic environment in lysosomes is maintained within a narrow pH range of 4.5–5 and plays an important role as a site of intracellular digestion. It is critical that the acidic pH is maintained since the hydrolases are inactive at greater pH values, resulting in the accumulation of protein aggregates and pathology.

**Figure 2:**
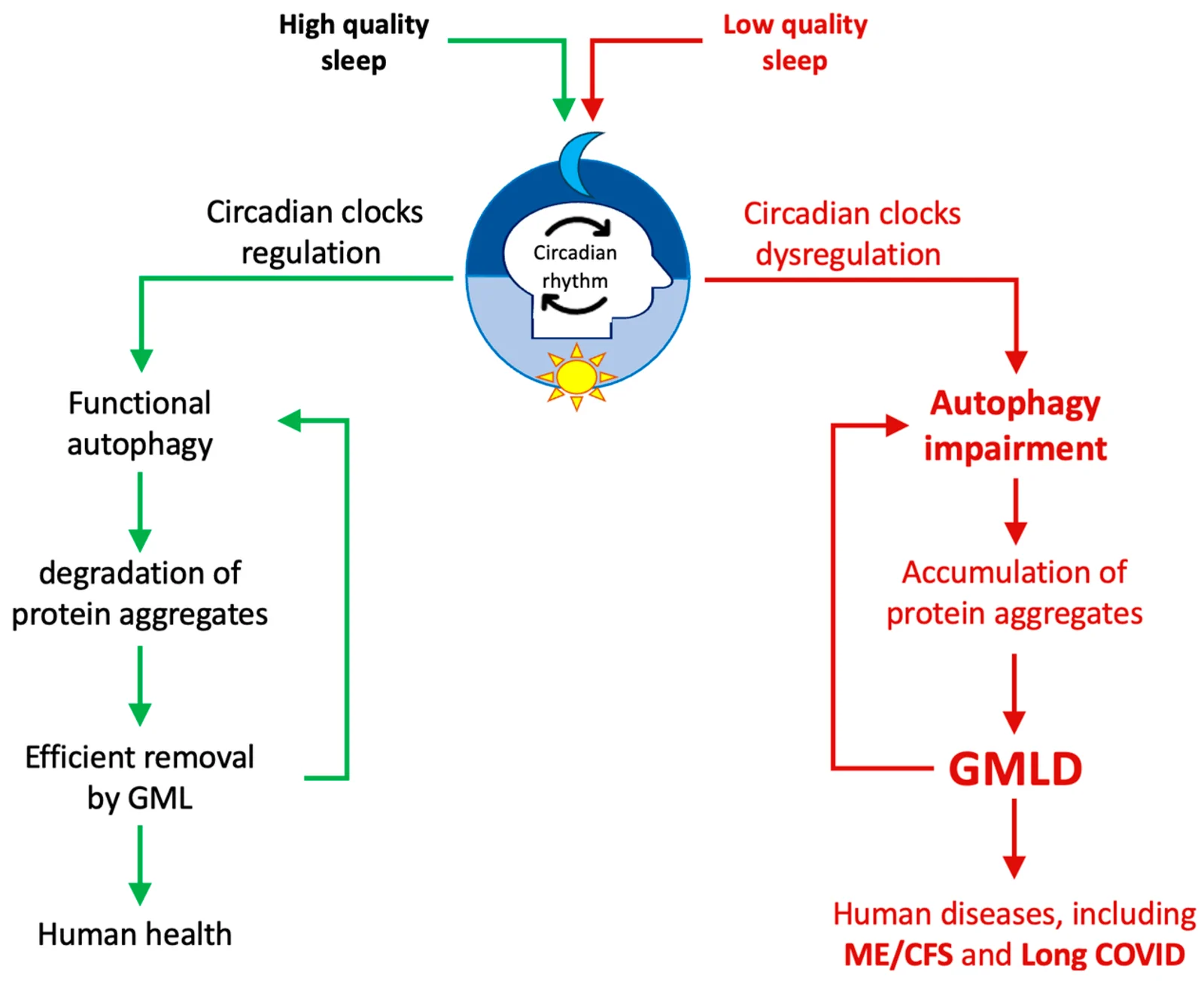
Putative relationship between autophagy, circadian clocks and sleep. While high-quality sleep can lead to efficient removal of aggregates, any sleep disturbances can significantly influence autophagy and cause glymphatic–meningeal lymphatic dysfunction (GMLD).

The glymphatic–meningeal lymphatic system and autophagy appear to operate as a cooperative waste clearance network, both exhibiting circadian rhythm in the brain. Autophagy is also activated by fasting; because sleep often coincides with fasting, it is the optimal time for this recycling process to occur (Figure 2).

While the fluid-based glymphatic–meningeal lymphatic system flushes the metabolic waste from the extracellular spaces surrounding the brain cells and out of the central nervous system, autophagy provides the complementary and final intracellular waste removal mechanism by breaking down and recycling the damaged cellular “trash” within cells. In this mechanism, lysosomes act as terminal organelles, responsible for degrading the materials delivered to them. Accordingly, the impairment of autophagy may potentially cause glymphatic dysfunction (GD), possibly as a result of the clogging of the perivascular spaces in the brain by damaged organelles and protein aggregates. Furthermore, autophagy impairment may cause astrocytes to become overloaded with these structures which has been described to play a role in the mislocalization (“depolarization”) of AQP4 water channels on astrocyte endfeet [50], with the possibility of restricting fluid transport by acting as a critical bottleneck, and inducing glymphatic failure.

During autophagy, autophagosome, a hallmark of autophagy, engulfs different protein aggregates, organelles and pathogens and then fuses with a lysosome to form an autolysosome. Inside, the hydrolytic lysosomal enzymes, such as cathepsins, break down the trapped cargo into basic reusable units. Accordingly, autophagy impairment is intimately connected to, and often caused by, the dysfunction of lysosomes. Any failure in the breaking down of damaged or misfolded proteins may lead to the accumulation of toxic protein aggregates, causing neurodegeneration or other pathologies, including cancer.

## 4. Autophagy, Circadian Rhythm and Sleep Dysfunction

Autophagy is a circadian process, and is generally more active during sleep when metabolic activities slow down in the course of the overnight fasting period in the brain, allowing cells to degrade damaged components and adjust to the different availability of energy supply. It can affect circadian rhythm both in normal and abnormal conditions in the brain and periphery [51], by degrading the circadian proteins. Accordingly, autophagy is regulated by sleep in a daily sleep–wake cycle (Figure 2).

Circadian clock proteins (e.g., BMAL1) regulate the expression of key autophagy genes to coordinate this cellular cleaning cycle with daily light/dark cycles [52]. Lack of sleep impairs sleep-driven autophagy, leading to a buildup of metabolic wastes such as amyloid aggregates (Figure 2). Unrefreshing sleep is a key hallmark of ME/CFS [53,54] and Long COVID [55,56], with a relationship that is likely bidirectional rather than unidirectional. While impaired autophagy and glymphatic clearance may contribute to poor sleep quality, sleep disturbances in ME/CFS and Long COVID may arise secondary to inflammation, autonomic dysfunction, metabolic abnormalities, or other disease processes, which could in turn further impair autophagy and glymphatic function (Figure 2).

During sleep, the glymphatic system is known to clear CNS metabolite wastes and other harmful substances that accumulate throughout daily brain activity [57], with astrocytes playing a major role [58]. The glymphatic system is primarily active during delta slow wave, non-rapid eye movement (NREM) sleep [57], when ME/CFS and Long COVID patients have greater sleep instability [59,60,61,62]. It is believed that autophagosomes accumulate during the day and are cleared at night [63], suggesting the close connection between autophagy and the glymphatic mechanism in the waste removal process. More interestingly, some studies suggest BMAL1 may influence autophagy and endolysosomal system in astrocytes, thereby playing an important role in astrocyte proteostasis and brain health [52].

## 5. Autophagy, Fatigue, Post-Exertional Malaise (PEM) and Pain

**Figure 3:**
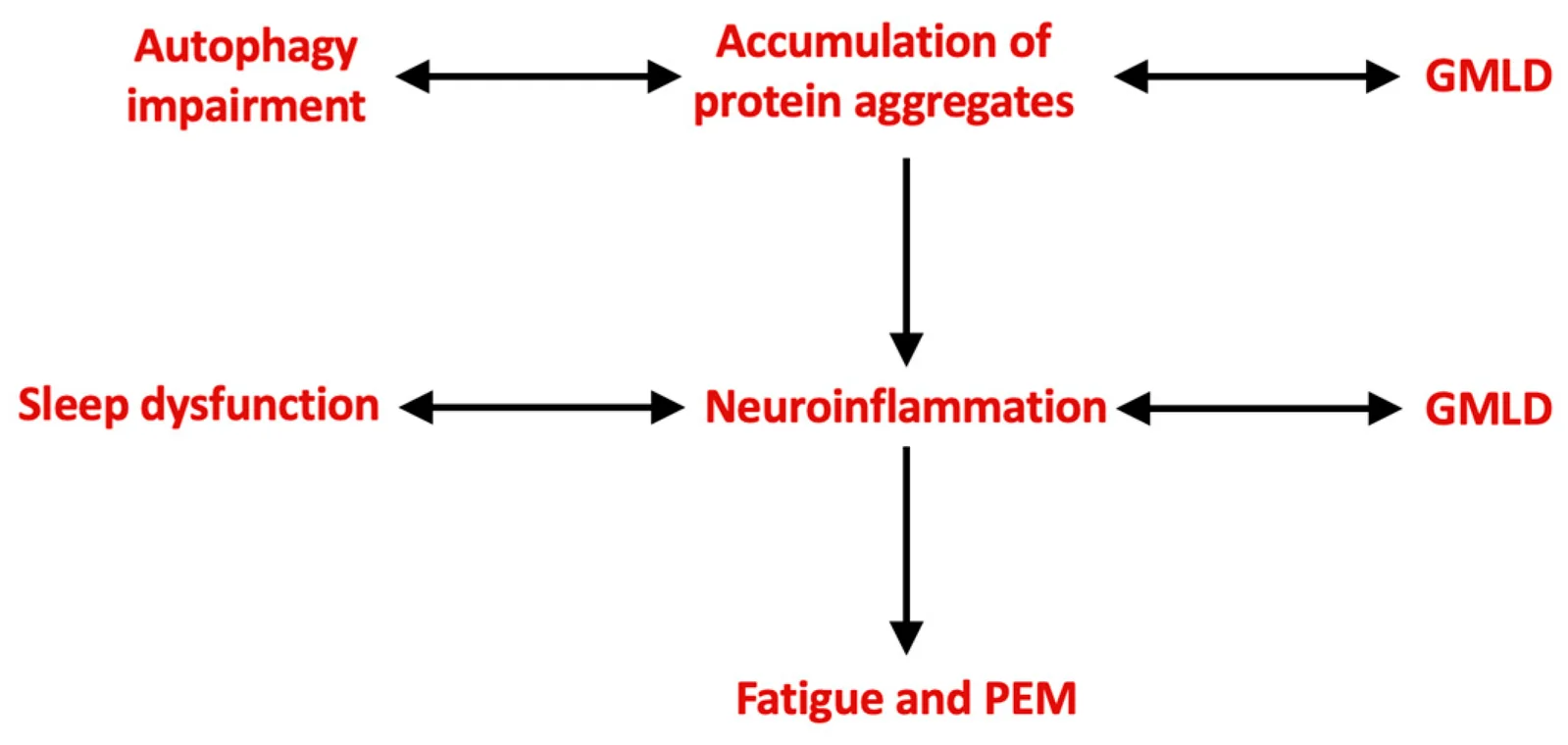
Proposed mechanisms by which neuroinflammation, mediated by autophagy impairment, may cause fatigue and PEM in ME/CFS and Long COVID; GMLD, glymphatic–meningeal lymphatic dysfunction.

In a recent study related to the pathophysiology of ME/CFS, correlations between impairment of autophagy and some of the key symptoms, including fatigue and PEM, have been described [64]. This is in accordance with an earlier study suggesting dysfunctional autophagy in this condition, with greater protein aggregation propensities in patient serum samples [17], in line with amyloid microclot formation, which have been reported for ME/CFS [18] and Long COVID [65]. The contribution of dysfunctional autophagy in promoting fatigue and PEM may be related to the induction of (neuro)inflammation, involved in various neurological diseases, including ME/CFS [66,67,68]. While several underlying mechanisms are suggested regarding the role of neuroinflammation in promoting fatigue, the dysregulation of neurotransmitter production and signaling, disturbance of sleep, induction of oxidative stress, and mitochondrial dysfunction have been suggested as some of the main mechanisms (Figure 3).

The lysosomal degradation pathway has been shown to involve the participation of molecular chaperones which have the capacity to assist protein folding, prevent aggregation, refold misfolded proteins, and aid in protein transport and degradation [69]. Chaperone-mediated autophagy is believed to rely entirely on these proteins to identify aggregates and shuttle them to lysosomes for destruction, thereby maintaining proteostasis. It has been suggested that ME/CFS patients may display a defective molecular chaperone response to strenuous exercise [70].

Molecular chaperones can be classified into two broad categories: ATP-dependent and ATP-independent [71]. For protein folding processes requiring the ATP-dependent chaperones, such as HSP 70 and HSP 90, ATP depletion can cause significant protein misfolding and aggregation. Accordingly, impaired mitochondrial synthesis of ATP in ME/CFS and Long COVID [72,73] can play an important role in this process.

Pain is a prominent symptom of ME/CFS, Long COVID and fibromyalgia [74,75,76,77]. Autophagy plays a critical role in modulating chronic and neuropathic pain [78,79] by reducing inflammation and clearing damaged nerve components. As discussed above, protein aggregate accumulation may cause autophagy impairment, GMLD and neuroinflammation (Figure 3), with the third cause being strongly related to the pathophysiology of neuropathic pain [80,81], chronic pain [82,83] and fibromyalgia [84,85].

## 6. Sources of Misfolded Proteins

**Figure 4:**
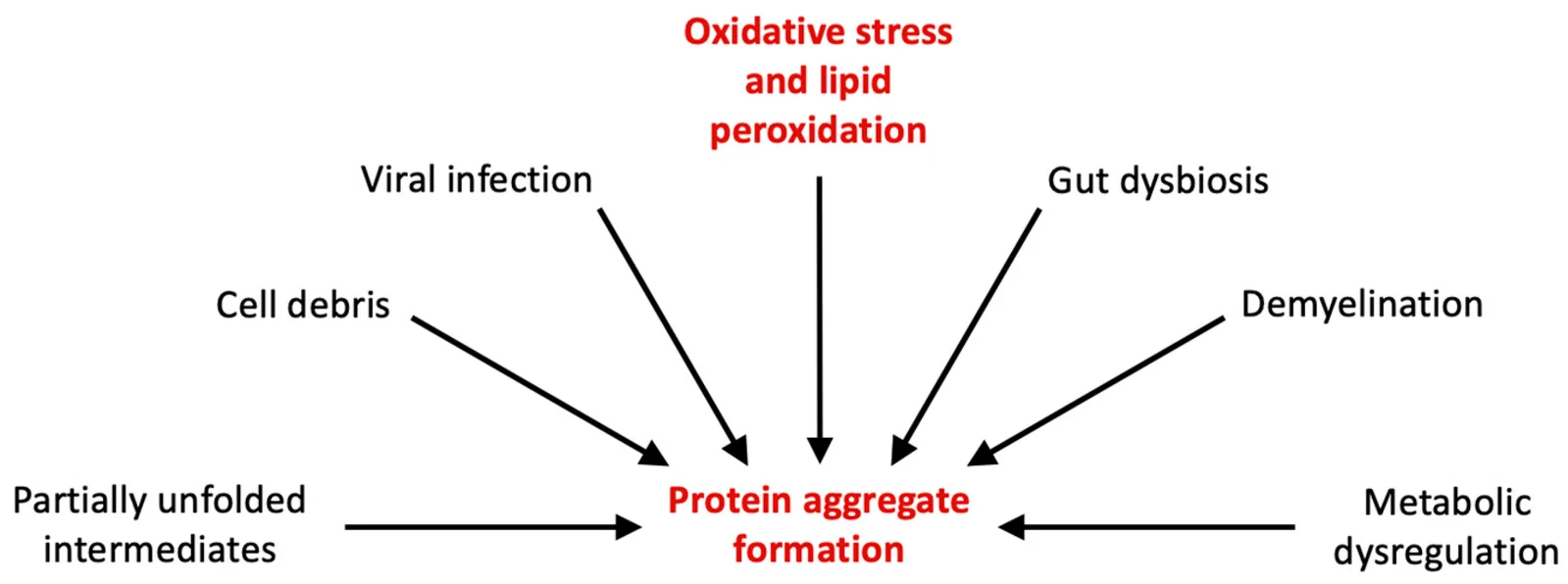
Some of the main sources of protein aggregate formation. The accumulation of protein aggregates can impair the normal function of both autophagy and the glymphatic–meningeal lymphatic system, leading to neuroinflammation.

Proteins are the most abundant biological macromolecules, making up over 50% of a cell’s dry mass and responsible for executing nearly all cellular tasks and vital functions. Linear polypeptide chains emerge from the ribosome and spontaneously undergo co-translational folding into functional three-dimensional structures, determined by their amino acid sequence. They ultimately take up a final structure which is more thermodynamically stable than the previously formed intermediate structures. Some of the intermediate structures can go through misfolding followed by formation of toxic aggregates, resulting in different pathologies, including neurodegeneration. Misfolded proteins may originate from several internal and external sources, including genetic mutations, cellular stress, environmental toxins, and infectious transmission. When these abnormal proteins evade the body’s quality control systems, they can accumulate and cause toxicity. There are a number of sources from where misfolded proteins may originate, some of which are indicated below (Figure 4).

### 6.1. Protein Aggregates from Intermediate Structures

The in vivo process of protein folding occurs during or after biosynthesis on ribosomes, with the polypeptides remaining unstructured during elongation, before taking up a native structure. Among these are the “molten-globular” forms, which arrange into native-like structures after emerging from the ribosome, with the tendency to aggregate [86]. It has been shown that the chemical modification and limited proteolysis of proteins may also cause formation of these or similar intermediate structures. Some of these structures have been identified to occur during the course of the amyloid fibrillization process, with specific significance related to neurodegenerative diseases [87].

Molten globule intermediate states have also been identified to occur from protein components of SARS-CoV-2 [88], and Epstein–Barr virus (EBV) [89], two neurotropic viruses implicated in the pathogenesis of Long COVID and ME/CFS [90].

### 6.2. Cell Debris

These are non-functional waste material left behind when cells rupture, die or degrade, and can be a source of misfolded protein aggregates. Cell debris in the brain has been described to include dead neurons, broken cell membranes and accumulated proteins, with microglia having a critical role in their clearance, involving autophagy [91]. Microglia activation, which can be triggered by misfolded proteins, is recognized as a primary cause of neuroinflammatory symptoms in ME/CFS [66] and Long COVID [92].

### 6.3. Senescent Cells

Senescent cells are damaged, growth-arrested cells that accumulate in tissue with age and are major sources of protein aggregates. Neurons, microglia, astrocytes and blood–brain barrier (BBB) endothelial cells are some of the senescent cell types in the brain. These are believed to be involved in the pathogenesis of Alzheimer’s disease, with senescence being induced by misfolded protein aggregates, resulting in neuroinflammation [93]. Furthermore, it has been suggested that addressing proteostasis disruption and senescence may offer the potential for Alzheimer’s disease treatment [93]. And by extension, we argue that these processes may play a role in ME/CFS and Long COVID.

### 6.4. Intracellular Pathogens

In ME/CFS, intracellular pathogens, such as EBV and *Coxiella burnetii*, may induce endoplasmic reticulum (ER) stress and disrupt cellular protein folding [94]. Subsequently, ER stress can lead to the overexpression and misfolding of host proteins, such as WASF3, that can ultimately impair mitochondrial energy production and cause exercise intolerance [95]. As for Long COVID, the production of prion-like protein aggregates has been described [96]. Also, the incubation of SARS-CoV-2 with human cerebrospinal fluid has been shown to induce amyloid aggregation of a number of proteins known to be involved in neurodegenerative diseases [97].

### 6.5. Gut Microbiome

The gut microbiota is a source of significant amyloid protein aggregates [98]. Dysbiosis in ME/CFS [99] and Long COVID [100] often leads to “leaky gut,” allowing bacterial amyloids and toxins into the bloodstream. It has been shown that exposure to amyloid proteins in the gut may cause priming of the immune system, eventually leading to the production of neuronal amyloid structures in the brain [101]. Additionally, misfolded proteins in the digestive tract can travel to the brain via the gut–brain axis, a bidirectional communication system between the brain and the digestive system. This is believed to involve the vagus nerve, with gut-derived misfolded protein aggregates causing pathology [102].

### 6.6. Axonal Damage/Demyelination

Demyelination has been reported for ME/CFS [7], and Long COVID [8,103], and the formation of protein aggregates in the brain and spinal fluid has been documented upon myelin basic protein damage [104]. This can potentially occur upon proteolytic digestion, involving catalytic autoantibodies [7]. More recently, such products have been described as prion-like misfolded protein aggregates with the capacity to cause neuronal damage [9], in line with the previous description of the neurodegenerative nature of this disease [105].

A number of studies have reported autophagy can enhance remyelination, and promote nerve regeneration, providing potential therapeutic approaches for the management of multiple sclerosis [106]. Moreover, autophagy modulation has provided potential therapeutic interventions for demyelination.

### 6.7. Oxidative Stress, Lipid Peroxidation and Protein Modification

Oxidative stress, a shared characteristic feature of ME/CFS and Long COVID [107], and lipid peroxidation occurring as the result, has been described to cause protein misfolding and aggregation [108,109]. Accordingly, lipid peroxidation is a common feature of neurodegenerative diseases [110], with its products occurring in the brains of both Parkinson’s and Alzheimer’s disease patients [111]. Also, oxidative stress can promote protein aggregation by their chemical modification [112]. In addition to membrane lipids, proteins may be a major target of oxidative modifications. Reactive species and their secondary products, including lipid peroxidation products, can oxidize proteins, leading to numerous types of protein post-translational modifications.

### 6.8. Fibrinogen-Resistant Microclots

Healthy endothelial cells normally help dissolve clots by secreting tissue-type plasminogen activator (tPA). During the endothelial dysfunction known to occur in ME/CFS [113] and Long COVID [114], they instead release an excess of plasminogen activator inhibitor-1, which has the ability to prevent blood clots from dissolving. Furthermore, it is believed that oxidative stress, a hallmark characteristic of ME/CFS and Long COVID [107], causes post-translational modifications (PTMs) that structurally alter fibrinogen. As the result of these events, instead of forming a normal, easily degraded fibrin mesh, the fibrinogen misfolds into amyloid-like, “fibrinaloid” microclot structures as reported for ME/CFS [18] and Long COVID [65], which are highly resistant to routine proteolytic breakdown. Some recent studies have suggested that these protein aggregates may cause the blockage of capillaries, which could potentially contribute to the reduced cerebral blood flow known to occur in ME/CFS [115], and Long COVID [116]. Autophagy is believed to be essential for maintaining vascular health and its role in endothelial function has been extensively documented (e.g., see [117]).

### 6.9. Metabolic Dysregulation

**Figure 5:**
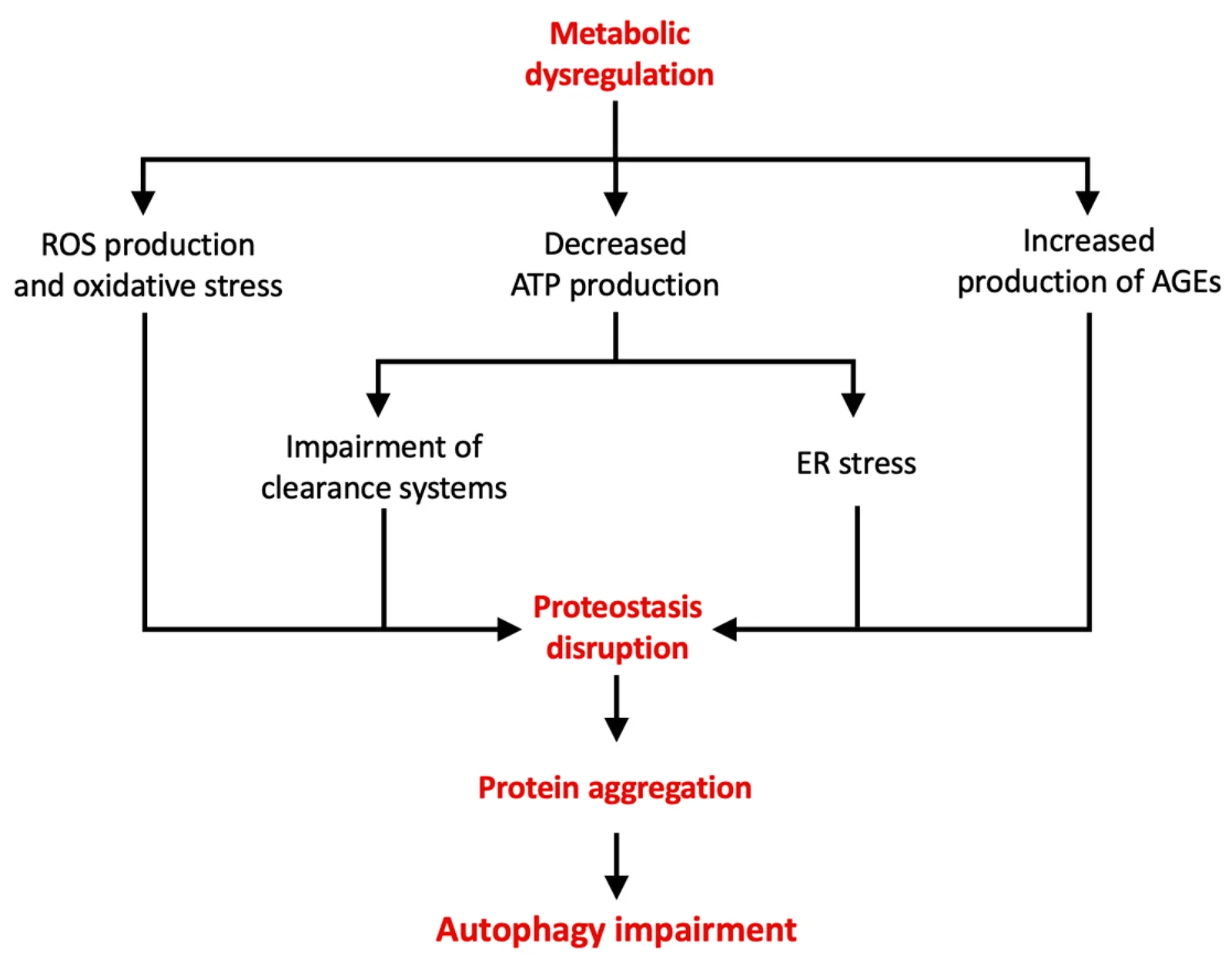
Some of the proposed mechanisms by which metabolic dysregulation may lead to protein proteostasis disruption in ME/CFS and Long COVID. This can promote the accumulation of protein aggregates and thereby cause a vicious cycle of autophagy impairment in these conditions. AGEs, advanced glycation end products.

Metabolic dysregulation has been abundantly reported for ME/CFS and Long COVID [118], and its role in causing protein misfolding and aggregation in neurological diseases has been documented [119]. The dysregulation of metabolic processes is believed to cause protein aggregation through several mechanisms (Figure 5).

For example, metabolic dysregulation can lead to decreased production of energy which is essential for the synthesis and proper folding of proteins in the ER. Moreover, metabolic dysregulation increases reactive oxygen species (ROS) production which can oxidatively modify proteins and thereby promotes protein misfolding and aggregation. Under conditions in which metabolic processes are dysregulated, the formation of advanced glycation end products (AGEs) can be increased, further exacerbating protein misfolding. Any disruption in the proteostasis network can affect the normal function of the endoplasmic reticulum, thereby leading to abnormal protein aggregation.

## 7. Types of Autophagy

After elucidating the main mechanistic features of starvation-induced, non-selective autophagy, commonly referred to as “bulk autophagy”, more recent focus has been directed toward exploring the roles of selective autophagy related to the degradation of specific targets. All different types of autophagy involve the formation of double-membraned vesicles, named autophagosomes, manipulated by proteins encoded by autophagy-related genes (ARGs) [120], more than 40 of which have been identified related to autophagosome formation. For the maintenance of cellular homeostasis, it is crucial that specific, harmful components are recognized and removed without the degradation of useful structures. Accordingly, this is a highly regulated process [121].

**Figure 6:**
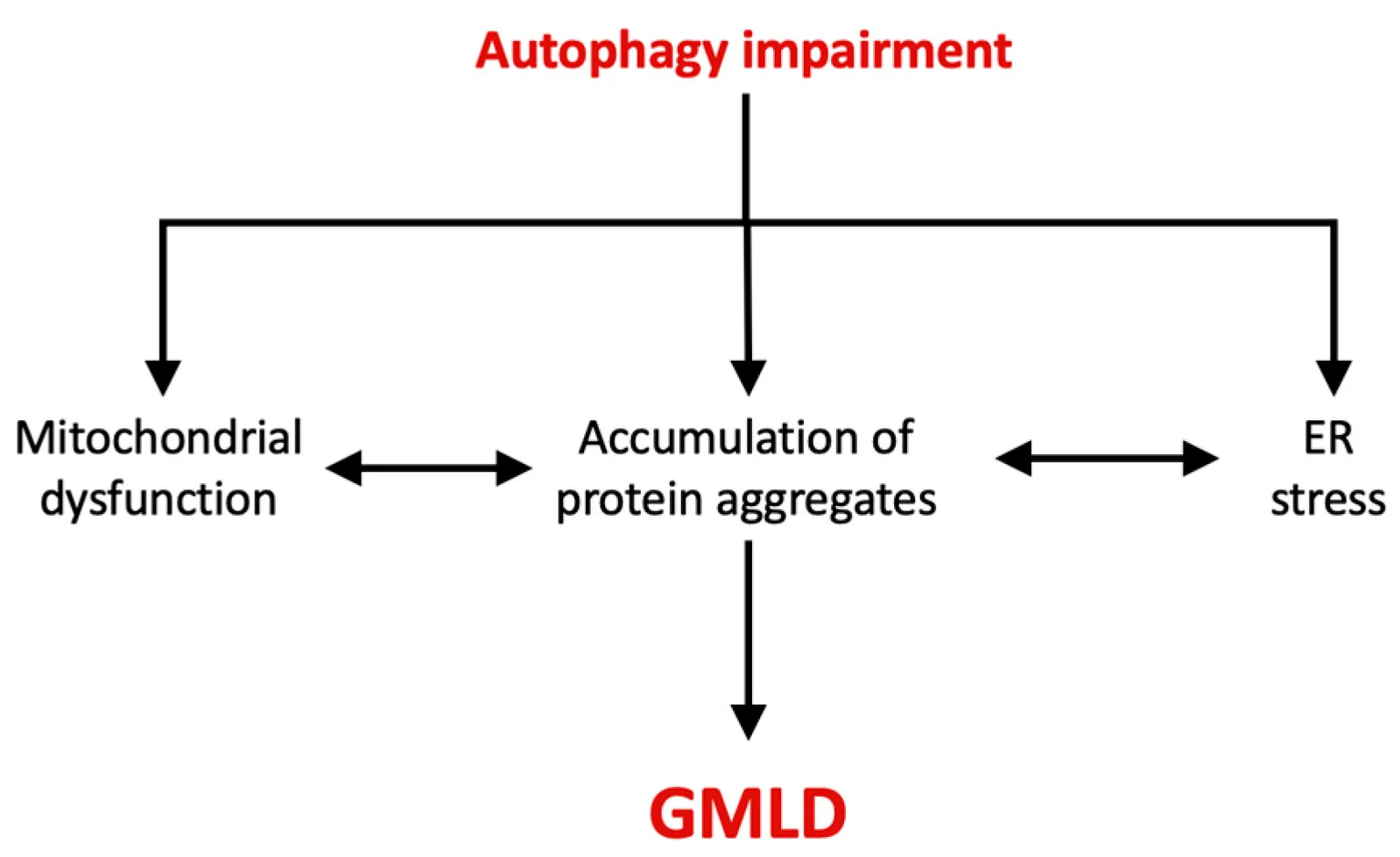
Proposed mechanisms by which autophagy impairment may cause GMLD in ME/CFS and Long COVID. GMLD, glymphatic–meningeal lymphatic dysfunction.

The following types of selective autophagy have been shown to continuously occur in the body to maintain cellular health. For most diseases, including ME/CFS and Long COVID, they are usually discussed under the general umbrella of “autophagy”, often without mentioning the specific types. Since all types of selective autophagy share much of the fundamental machinery, it is believed that a defect in the pathway can disrupt the cell’s ability to clear a wide variety of specific targets. Below, we discuss the different types of selective autophagy, which have been reported to be involved in the molecular pathophysiology of ME/CFS and Long COVID (Figure 6).

### 7.1. Aggrephagy

Misfolded proteins are prone to aggregation owing to the presence of unstructured regions. Aggrephagy is the process by which cells selectively target, encapsulate, and break down toxic or misfolded protein aggregates (aggresomes) through autophagy [122]. Accordingly, aggrephagy acts as a vital cellular quality control system, clearing out intracellular debris that would otherwise disrupt normal function and lead to pathology (e.g., ref. [123]). Misfolded protein aggregates consisting of amyloid fibrin(-ogen) have been observed in ME/CFS and Long COVID [18,19,20], and ME/CFS serum samples have been described to display “a greater aggregation propensity” [17]. This further suggests the occurrence of an impaired aggrephagy, and that misfolded protein aggregates may play an important role in their pathophysiology.

### 7.2. Mitophagy

Mitophagy is a specialized form of autophagy that maintains mitochondrial functional integrity and cellular homeostasis by specifically removing dysfunctional mitochondria [124]. It is a critical process in ME/CFS and Long COVID, where mitochondrial dysfunction is believed to occur [72,125].

Defects in the respiratory chain complex proteins can result in energy insufficiency and the accumulation of reactive oxygen species reported in these conditions [107]. Therefore, in order to prevent accumulation of impaired mitochondria, they must be selectively degraded.

Mitophagy and the glymphatic system are both believed to be critical for maintaining brain health [126], processes which can be impaired in neurological diseases like Alzheimer’s and Parkinson’s, leading to the accumulation of harmful protein aggregates and brain damage.

### 7.3. ER-Associated Autophagy: ER-Phagy

This is a specific pathway that traffics misfolded proteins directly from the endoplasmic reticulum (ER) to lysosomes [127]. It is responsible for maintaining ER health, quality control, and function, especially during stress, like a buildup of misfolded protein or starvation, by removing damaged ER parts. This process is crucial for cellular homeostasis, responding to ER stress, which is believed to be a primary driver of autophagy failure. Furthermore, it has been reported that ER stress upregulates the WASF3 protein (Wiskott–Aldrich Syndrome Protein Family Member 3), which subsequently disrupts normal mitochondrial respiration and triggers cellular exhaustion, in ME/CFS and Long COVID [95,128].

### 7.4. Xenophagy

Xenophagy is an evolutionarily conserved immune mechanism responsible for the elimination of intracellular pathogens, thereby defending against bacteria, viruses (virophagy) and parasites, invading inside the host cells [129]. It not only removes pathogens but also contributes to innate and adaptive immunity by facilitating the presentation of antigens to immune cells. Virophagy [130] is of special significance for ME/CFS and Long COVID as post-infectious diseases and has been described to play a special role in controlling SARS-CoV-2 infection [131].

### 7.5. Pexophagy

Peroxisomes are highly metabolic organelles that both generate and scavenge reactive oxygen species. Consequently, cells must maintain peroxisome homeostasis which is achieved by this special type of autophagy [132] to remove damaged peroxisomes and maintain cellular health. Accordingly, this selective autophagy impairment may lead to the accumulation of malfunctioning peroxisomes and pathology, including neurological diseases. Moreover, oxidative stress which has been shown to be caused by malfunctioning peroxisomes is an important part of the pathophysiology of ME/CFS and Long COVID [107].

### 7.6. Ferritinophagy

Ferritinophagy is a selective type of autophagy in which cells degrade ferritin—the primary iron-storage protein—to recycle iron and maintain intracellular iron homeostasis [133]. It causes the release of ferrous ions (Fe^2+^) into the cytosol, thereby fulfilling the cell’s metabolic needs for this ion. Accordingly, higher-than-normal levels of serum ferritin (hyperferritinemia) can indicate autophagy impairment, leading to cellular iron deficiency. The role of ferritinophagy in a number of pathologies, including cardiovascular, autoimmune and neurological diseases, has been investigated [134]. Elevated ferritin is increasingly recognized as a key biomarker in some ME/CFS patients, particularly for those with post-infectious onset like Long COVID [135,136]. A recent animal-model study found that pseudorabies virus, an alphaherpesvirus used to model viral encephalitis, triggers ferritinophagy-mediated ferroptosis and neuroinflammation [137]; whether this mechanism extends to the viruses implicated in ME/CFS and Long COVID has not yet been tested, though it may be mechanistically relevant to their pathophysiology [66,138,139].

### 7.7. Lipophagy

Lysosomes break down and recycle intracellular lipid droplets through a process called lipophagy. It is a critical mechanism for maintaining cellular energy balance, recycling lipids, and preventing lipotoxicity. Lipid droplets are organelles that serve as lipid reservoirs in many cell types, and the accumulation of lipids in cells often indicates this type of autophagy impairment [140], which has been investigated related to various pathologies, including neurological dysfunctions. The accumulation of lipid droplets in cells has been reported to occur in ME/CFS [141], and COVID-19 [142].

### 7.8. Glycophagy

Glycogen, a multibranched polysaccharide comprised of glucose, is a major form of energy storage in muscle and liver cells, and its regulation plays a crucial role in energy metabolism, which can be of importance related to ME/CFS and Long COVID. The breaking down of glycogen to glucose, termed glycogenolysis, is mediated by glycogen phosphorylase. Additionally, cells can break down glycogen through autophagic degradation, a process termed glycophagy [143]. This form of autophagy has been investigated for various pathologies, and as a potential therapeutic target [144]. Brain glycogen is stored mainly or exclusively in astrocytes, depending on the situation, in which glycophagy has been shown to play an important role [145].

## 8. Autophagy in Specific Cell Types and Its Significance

**Figure 7:**
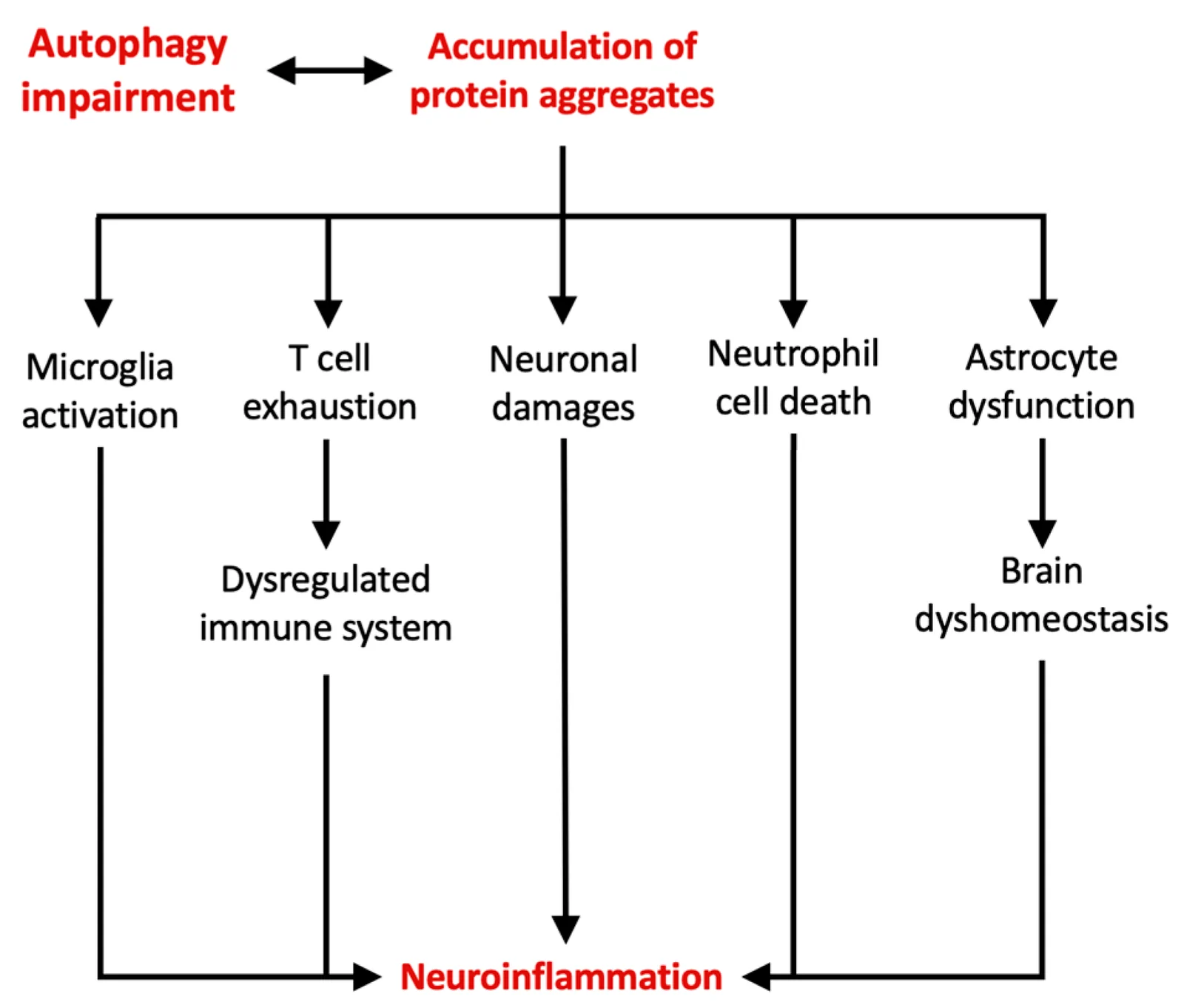
Neuronal cell toxicity induced by protein aggregates. Accumulation of protein aggregates mediated by autophagy impairment can damage various cell types, leading to neuroinflammation.

Cells maintain health by clearing out damaged organelles, misfolded proteins and pathogens, and may use autophagy as a major intracellular degradation system. Accordingly, the accumulation of misfolded protein aggregates is believed to be a direct cause of pathology in numerous cell types across a wide range of human diseases. Below, we discuss how autophagy may play a role in maintaining cell homeostasis in neurons and four other cell types in ME/CFS and Long COVID (Figure 7):

### 8.1. Neurons

Since neurons are post-mitotic (terminally differentiated) and exit the cell cycle, they cannot divide to dilute out toxic metabolic wastes across daughter cells, a mechanism frequently used by proliferating cell types. Accordingly, they are uniquely sensitive to toxic protein accumulation and are believed to be heavily dependent on continuous proteostasis, including autophagy [146]. Moreover, neurons are highly polarized, consisting of complex networks of dendrites and long axons, with lysosomes being most abundant in their cell bodies. This means protein aggregates must be transported long distances for efficient degradation, again providing the opportunities for causing toxicity. Neuroinflammation occurring as a result is believed to contribute to the pathophysiology of ME/CFS and Long COVID [66,138,139]. Also, by damaging neurons in the central and peripheral nervous system, misfolded proteins can cause chronic and neuropathic pain, as discussed related to ME/CFS, Long COVID and fibromyalgia in Section 5, above.

### 8.2. Astrocytes

Astrocytes play a critical role in the glymphatic function. They are prone to misfolded protein accumulation, and consequently dysfunctional lysosomes in these glial cells may contribute to neuronal death [147]. It has been reported that autophagy in astrocytes provides a crucial cellular clearance and recycling mechanism for maintaining brain homeostasis [148].

Astrocytes are believed to actively contribute to the removal of extracellular protein aggregates in the brain and have been considered as potential therapeutic targets [149]. Defective autophagy in these glial cells may lead to the accumulation of protein aggregates, clogging the brain’s perivascular spaces, and affecting the glymphatic system [11].

The role of astrocytes in dealing with pathological prion-like protein aggregates in neurodegeneration (proteinopathies) has also been described [150], and the circadian clock protein BMAL1 has been reported to influence autophagy in these cells [52]. This corresponds to the circadian nature of autophagy and glymphatic system, with potential significance in sleep dysfunction in ME/CFS [60] and Long COVID [151]. Also, astrocyte dysfunction by misfolded proteins can drive chronic and neuropathic pain [152,153].

### 8.3. Microglia

Microglia are prone to misfolded protein accumulation [154] and have the ability to transport them to neighboring neurons and other glial cells, with autophagy believed to be involved in their clearance. Accordingly, enhancing this process has been suggested to provide a promising therapeutic strategy in neurological diseases, including Alzheimer’s disease. More specifically, autophagy has been shown to enable microglia to be neuroprotective by removing amyloid β protein (Aβ), a major component of extracellular plaques found in Alzeimer’s disease, thereby preventing microglia senescence [155].

A number of studies have reported neuroinflammation occurring in ME/CFS [66,138] and Long COVID [139], with the involvement of microglia activation [138], which can be caused by misfolded protein aggregates such as Aβ in these cells. Moreover, the clearance of these structures is afforded by the glymphatic system [156], the dysfunction of which is also connected to neuroinflammation [157]. Microglia activation results in the release of pro-inflammatory cytokines, such as IL-1β and TNF-α, that disrupt and damage surrounding neurons and is believed to play a major role in the pathophysiology of fibromyalgia [84,158].

### 8.4. T Cells

A toxic buildup of misfolded proteins may cause “T cell exhaustion” [159], widely recognized as an important feature of ME/CFS and Long COVID [160,161]. However, the role of misfolded proteins in causing T cell exhaustion in these diseases has not been described. This is a state of immune dysfunction where T cells—particularly CD8+ “killer” T cells—lose their ability to function effectively. Autophagy is believed to be a crucial contributor to T cell survival, metabolism, and functional longevity. It is a major contributor to maintaining T cell homeostasis and is believed to help prevent exhaustion by removing damaged organelles and toxic metabolic by-products. When autophagy is inhibited (e.g., in high lipid environments), T cells show reduced proliferation and defective cytokine production, characteristic of an exhausted state [162].

### 8.5. Neutrophils

Neutrophils are the most abundant type of white blood cells and are regarded as the first line of defense in the innate immune system, performing a range of immune functions, including phagocytosis and autophagy [163] to target, capture, and digest invading pathogens. It has been shown that misfolded protein aggregates can cause apoptosis [164], reported to be increased in ME/CFS [165].

## 9. Factors Enhancing Protein Misfolding and Aggregation

Some of the factors that can induce protein misfolding and aggregation are listed below:

### 9.1. Lipopolysaccharides

Lipopolysaccharides (LPS) and some other bacterial endotoxins are implicated in the pathogenesis of ME/CFS [166], and Long COVID [167]. They are anionic amphipathic (amphiphilic) polymers that interact with proteins, induce misfolding and accelerate the aggregation process [168]. This is the consequence of compromised intestinal barrier in these diseases pointed out above, allowing bacteria to leak into the bloodstream. Also, LPS can break down the blood–brain barrier, thereby entering the brain parenchyma, where it may facilitate the accumulation of amyloid plaques and other misfolded protein aggregates [169]. These structures can cause neuroinflammation, which have been described for ME/CFS and Long COVID [66]. Also, LPS by itself, may directly cause autophagy impairment [170].

### 9.2. Cross-Seeding Involving Other Protein Aggregates

Aggregated proteins have been shown to behave as templates that induce misfolding of other proteins, a process known as templated conformational conversion or “seeding”. When protein aggregates come into contact with a correctly folded protein, they can force such proteins to unfold and these newly misfolded proteins may then join a growing aggregate and accelerate (“catalyze”) the formation and spreading of other misfolded proteins throughout the cell or tissue [171]. This is frequently described as a “prion-like” mechanism which is the underlying cause of several neurodegenerative diseases [172].

### 9.3. Seeding Involving Surface-Assisted Catalysis by Viruses

Neurotropic viruses, such as EBV, cytomegalovirus (CMV) and the SARS-CoV-2 virus are believed to be linked to neurodegenerative diseases by acting as catalytic surfaces that can convert soluble proteins into amyloid aggregates. This has been described as a surface-assisted cross-seeding mechanism for these viruses, including EBV [173] and SARS-CoV-2 [174,175,176], suggesting possible long-term complications. Also, some components derived from these and other neurotropic viruses, may catalyze formation of large protein aggregates from aggregation-prone proteins (e.g., α-synuclein, tau, and TDP-43 [177]) in the brain [23], and cerebrospinal fluid [97].

## 10. “Infectivity”

The concept that a protein can be “infectious” was first demonstrated by Stanley Prusiner, when he, together with his co-workers, purified the prion protein responsible for scrapie [178]. Later, in 1997, he was awarded the Nobel Prize in Physiology or Medicine [179]. Since this discovery, the infectious nature of prions—their ability to cause the misfolding of neighboring proteins with native structures—has been extensively documented as a novel biological principle of infection [180]. Accordingly, it is now well-established that prions are completely devoid of nucleic acids and represent a unique class of infectious agents. By acting as self-propagating templates (seeds), they can cause the misfolding of other proteins, resulting in neurodegenerative diseases [181]. Accordingly, this unique templated autocatalytic process, usually referred to as “prion-like”, is believed to provide a mechanism of fundamental importance to protein aggregation in a number of well-characterized neurodegenerative conditions, including Alzheimer’s disease, Parkinson’s disease and amyotrophic lateral sclerosis.

The spike protein in SARS-CoV-2 has been described to contain prion-like domains causing neurological problems [182], with proinflammatory properties and aggregation potency. Furthermore, as indicated above, the amyloid-like fibrinoid microclots reported for ME/CFS [18], and Long COVID [65], have been described to resemble prion proteins [65], and a recent study has pointed out how the spike protein of SARS-CoV-2 would contribute to the formation of such amyloid microclots [183]. There have been extensive discussions on the role of autophagy in prion diseases (e.g., see [184]), the pharmacological activation of which, as a neuronal defensive strategy, has been investigated [185].

## 11. Cellular Toxicity of Misfolded Proteins at the Membrane Level

Protein oligomers can interact with biological membranes, including plasma, lysosomal and mitochondrial membranes, causing membrane disruption [186,187], pore formation [188], and ultimately permeabilization [186], mainly through a detergent-like mechanism [186,189,190,191]. In Parkinson’s disease and Alzheimer’s disease, α-synuclein (α-Syn) and amyloid-β (Aβ) aggregates may cause disruption of neuronal membrane upon insertion into the lipid bilayer [192]. These aggregates can damage synapses and dysregulate their function, leading to ion dyshomeostasis and neurotransmitter signaling dysregulation [193].

Lysosomal membrane integrity is of paramount importance for maintaining autophagy [194]. Various misfolded protein aggregates have been shown to cause the disruption of lysosomal membranes [195], which can result in the release of lysosomal enzymes into the cytosol, accompanied by AI and triggering cell death pathways [196]. Also, the integrity of lysosomal membrane is essential for mTORC1 binding and activity [197].

**Figure 8:**
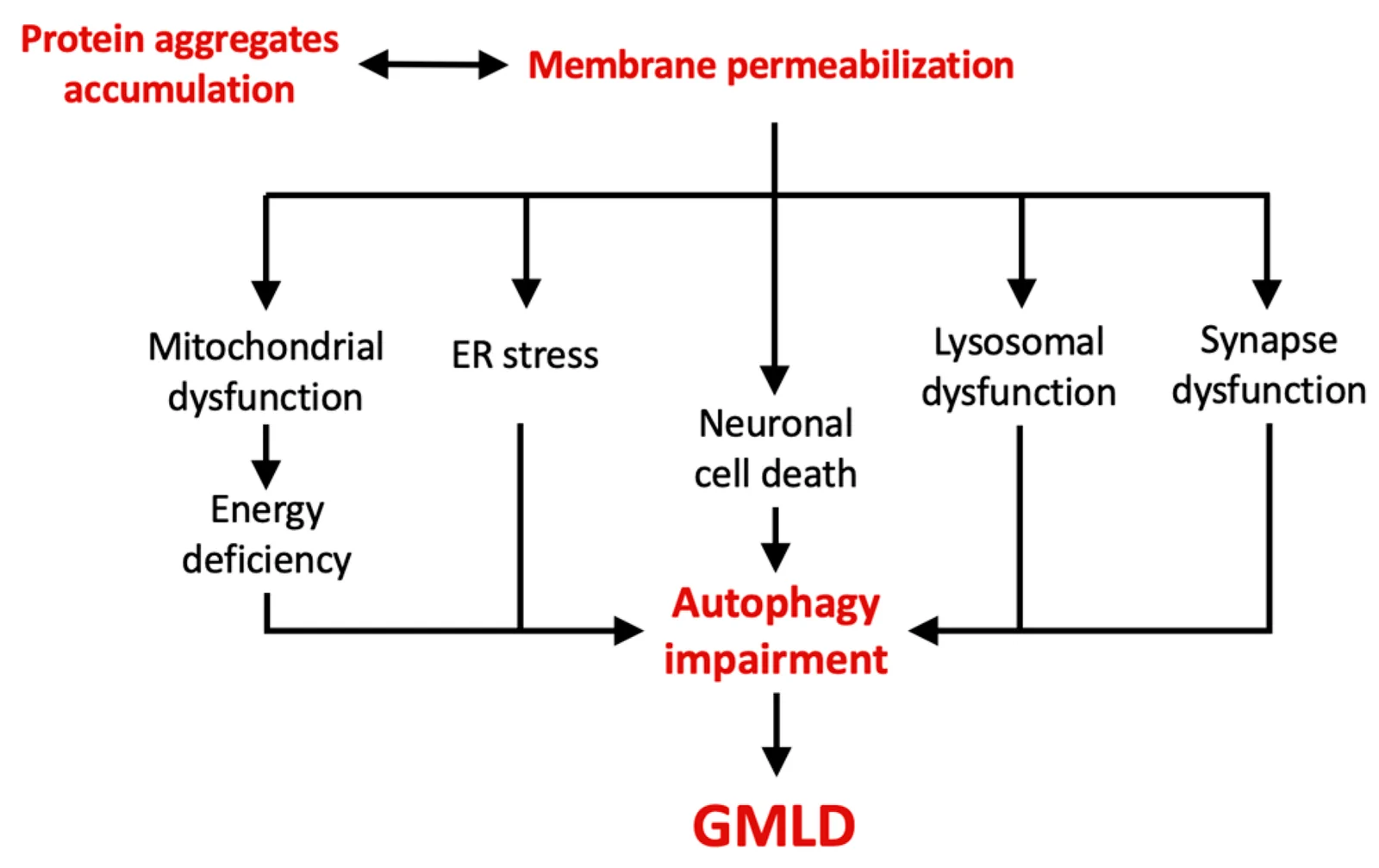
Proposed mechanisms describing how membrane permeabilization mediated by protein aggregates may cause autophagy impairment and GMLD (glymphatic–meningeal lymphatic dysfunction). Various biological membranes may be affected by the general “detergent-like effects” of the amphiphilic misfolded protein structures, as described above.

In mitochondria, misfolded proteins may induce toxicity by direct interaction with membrane proteins or phospholipids, leading to the alteration of their function and bioenergetic failure which can affect chaperone-mediated autophagy as discussed above. Any autophagy impairment may promote the accumulation of protein aggregates and may lead to a vicious cycle that may further impair the glymphatic clearance system (Figure 8).

The endoplasmic reticulum is very important in managing misfolded proteins by activating the unfolded protein response (UPR). Accumulation of protein aggregates, including amyloid beta and alpha-synuclein, are known to impair normal function of the ER [198]. These can significantly influence the proteostasis network, which is responsible for normal protein folding, trafficking and degradation, leading to exacerbating protein misfolding and aggregation (Figure 8).

## 12. Ammonia Dysregulation and Autophagy

Autophagy facilitates ammonia detoxification through cooperation with the urea cycle toward ammonia homeostasis [199], the dysregulation of which has been implicated in ME/CFS and Long COVID [118]. Accordingly, a dysfunctional autophagy may contribute to hyperammonemia, a well-established cause of neuroinflammation [200], which occurs in ME/CFS and Long COVID [201], in addition to causing glymphatic dysfunction [66,202].

## 13. Environmental Factors as Potential Causes of Autophagy Impairment

A number of environmental factors, including chemicals, heavy metals and toxins, implicated in the pathophysiology of ME/CFS and Long COVID have been found to cause autophagy impairment (e.g., ref. [203]). Also, many pesticides have been found to cause the impairment of autophagy (e.g., ref. [204]). Some of these chemicals can bring about lysosomal membrane permeabilization, as indicated in the case of malathion [205], or cause direct inhibition of lysosomal enzymes by binding to their (hydrophobic) active sites (e.g., ref. [206]).

## 14. Autophagy, Oxidative Stress, Inflammation and Autoimmunity

### 14.1. Oxidative Stress

**Figure 9:**
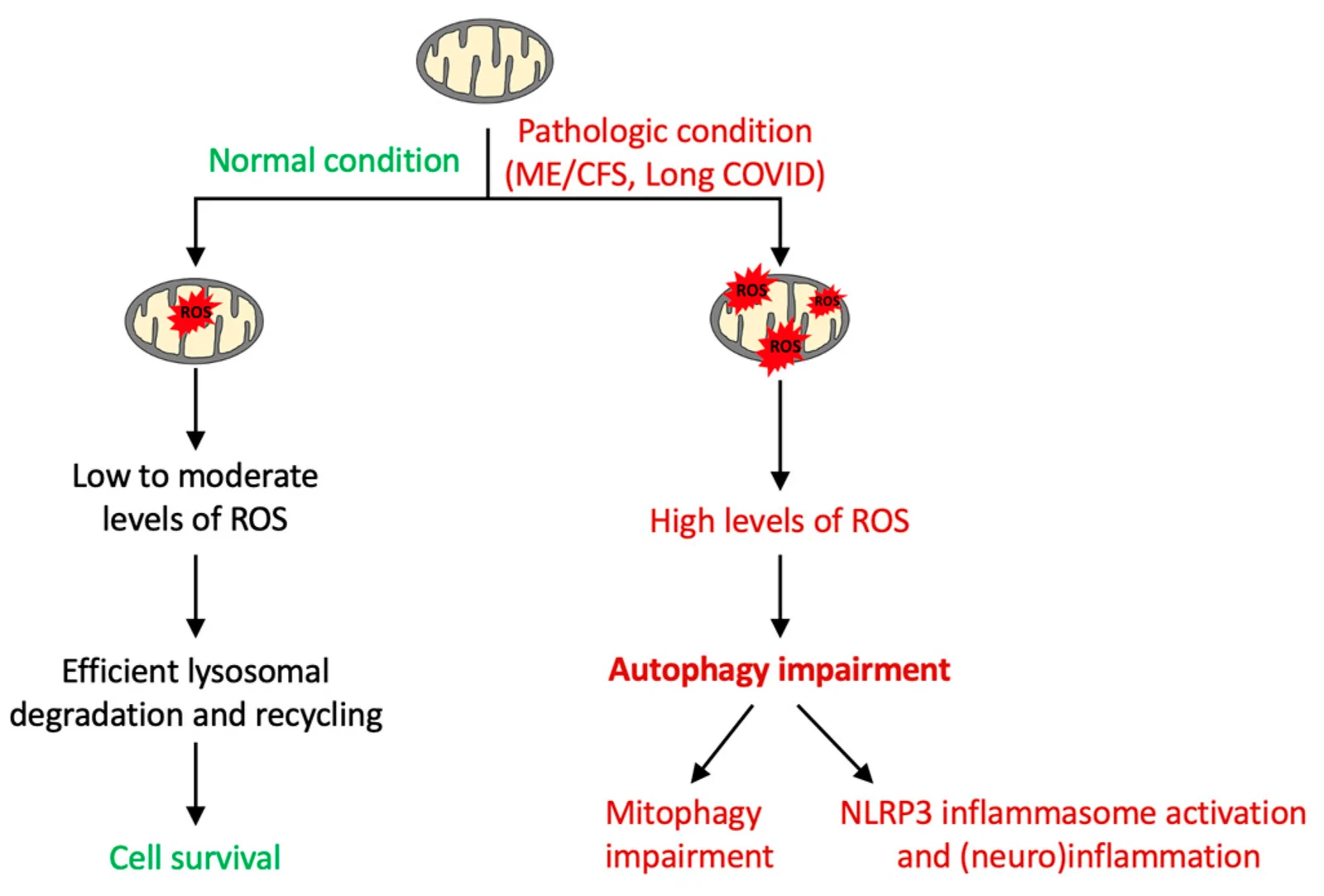
A potential role of mitochondrial ROS in the regulation of autophagy. While low levels of ROS are essential for the efficient degradation and removal of protein aggregates and damaged components, high levels of mitochondrial ROS impair autophagy and cause mitophagy impairment and uncontrolled activation of the NLRP3 inflammasome.

Oxidative stress may act as one of the main modulators (e.g., ref. [207]). While low levels of ROS can trigger autophagy, helping cells adapt and survive under stress by clearing damaged organelles and proteins, excessive or chronic oxidative stress can disrupt autophagy and cause pathology (Figure 9).

Mitochondria are widely recognized as a major subcellular producer of ROS required for autophagy induction [208]. Accordingly, mitochondrial dysfunction in ME/CFS and Long COVID [125,209] may be connected with autophagy (Figure 9). Furthermore, oxidative stress can damage lysosomal membrane integrity by inducing the cross-linking of lysosome membrane proteins via the formation of disulfide bonds [210], resulting in the leakage of lysosomal enzymes and inducing a vicious cycle of events as depicted in Figure 9. Accordingly, the general understanding is that oxidative stress can lead to the dysregulation of autophagy, either by its inhibition or overactivation. Both of these outcomes can contribute to cell damage and disease, providing therapeutic opportunities, with some antioxidants being effective [211].

### 14.2. Inflammation

Inflammation has been described to be a cause of protein misfolding and aggregation [212]. The interaction between inflammasomes and autophagy is believed crucial for maintaining the balance between optimal immune responses and the avoidance of excessive inflammation [213]. Autophagy machinery (e.g., autophagosome) can capture ubiquitinated inflammasome components (such as NLRP3) for degradation and removal [214]. Also, it can inhibit the activation of the NLRP3 by targeting damaged mitochondria (mitophagy) that produce ROS and inflammasome activation, as a result (Figure 9) [215]. Autophagy has also been studied in relation to neuroinflammation reduction in neurological conditions, which can be of potential benefit to ME/CFS and Long COVID patients who also suffer from neuroinflammation [66,139,216,217].

### 14.3. Autoimmunity

Numerous studies have reported that autophagy is heavily involved in immune regulation (see, e.g., ref. [218]), with its dysfunction being related to various autoimmune diseases [219]. Additionally, protein misfolding and aggregation may trigger autoimmunity [220]. This mechanism may be important related to ME/CFS and Long COVID, which are believed to have an autoimmune component in their pathophysiology.

## 15. Dysregulated Autophagy Related to Symptoms/Dysfunctions in ME/CFS and Long COVID

**Figure 10:**
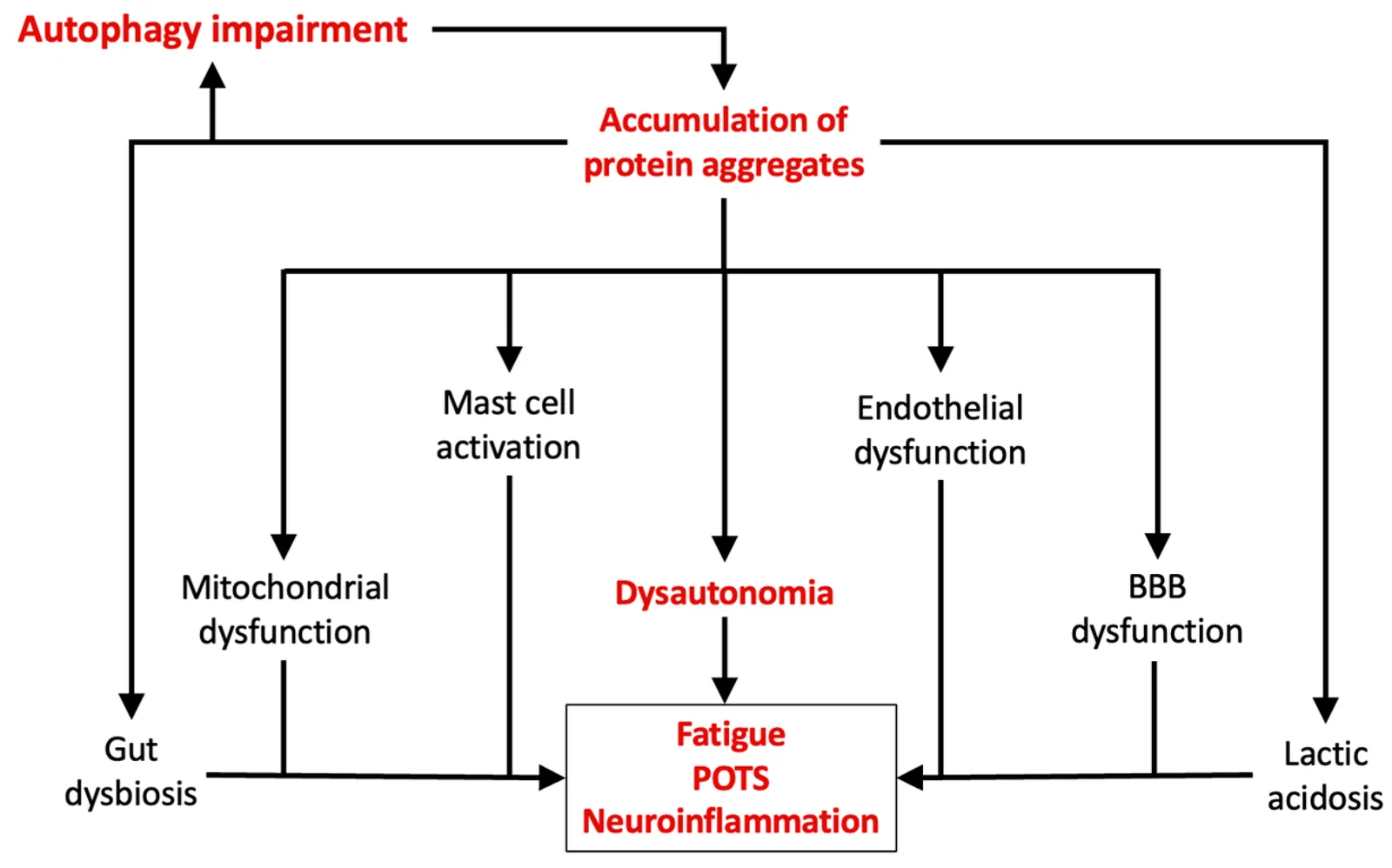
Proposed mechanisms by which accumulation of protein aggregates mediated by autophagy impairment may lead to fatigue, postural orthostatic tachycardia syndrome (POTS) and neuroinflammation.

Autophagy impairment has been linked to a broad range of human pathologies including neurological diseases, cancer and metabolic diseases. Some of the related mechanisms are described below for ME/CFS and Long COVID (Figure 10).

### 15.1. Neurological Dysfunctions

A number of studies have described the role of dysregulated autophagy in various neurodegenerative conditions (e.g., ref. [221]), which are also of significance related to ME/CFS and Long COVID, both of which have been reported to have neurological symptoms.

### 15.2. Endothelial Dysfunction

Autophagy is believed to contribute to maintaining endothelial health [117], and its dysregulation can be damaging, potentially contributing to the endothelial dysfunction occurring in ME/CFS [113], and Long COVID [114]. The misfolded protein aggregates in the form of amyloid microclots observed in ME/CFS [18] and Long COVID [65] can contribute to endothelial dysfunction, and an active autophagy would play a critical role in their removal.

### 15.3. Blood–Brain Barrier (BBB) Dysfunction

BBB is a selective semipermeable membrane that plays a crucial role in maintaining CNS homeostasis, which may be affected by misfolded proteins [222]. In addition to its ability to cause the degradation of these aggregates, autophagy is believed to play an important role in protecting BBB under various conditions, including oxidative and hypoxic stress. Also, autophagy in astrocytes is believed to cause the removal of misfolded proteins developed in these cells [48], thereby preventing the blockage of aquaporin-4 (AQP4) water channels by these aggregates, which could otherwise impair fluid movement across the BBB. BBB dysfunction has been described in ME/CFS [3,66] and Long COVID [223].

### 15.4. Mitochondrial Dysfunction and Fragmentation

Two of the main consequences usually discussed in relation to the detrimental effects of misfolded proteins on mitochondrial homeostasis are mitochondrial dysfunction [224] and fragmentation [225], which have been reported to occur in ME/CFS [72] and Long COVID [125]. In addition to misfolded proteins causing membrane pore formation and permeabilization discussed above, they can cause the release of cytochrome c and mitochondrial enzymes from the inner and outer mitochondrial membranes [186], and impairment of the activity of the matrix enzymes and complexes of the electron transport chain.

### 15.5. Mast Cell Activation

Mast cell activation syndrome is now believed to be an important component of the pathophysiology of ME/CFS and Long COVID, where persistent inflammation coincides with abnormally high mast cell activation. Accordingly, it has been extensively documented for ME/CFS (e.g., ref. [226]) and Long COVID (e.g., ref. [227]), with many patients believed to benefit from targeted therapies. Misfolded protein aggregates, such as amyloid beta peptides, have been described to interact with mast cells, causing their activation [228].

### 15.6. Platelet Hyperactivation

ME/CFS [18] and Long COVID [229] have been reported to be accompanied by substantial platelet activation. Misfolded protein aggregates, including β-amyloid, and amyloid fibrin microclots, which frequently develop in ME/CFS and Long COVID, have been found to interact with platelets and cause their activation [18,230].

### 15.7. Apoptosis

Maintaining cellular homeostasis is believed to rely on the interplay between autophagy and apoptosis [231]. Misfolded protein aggregates can trigger apoptosis when they accumulate by different mechanisms, including through chronic, unresolved endoplasmic reticulum (ER) stress, known as the unfolded protein response (UPR) [232], and release of cytochrome c from the inner mitochondrial membrane. Accordingly, a dysfunctional autophagy may be one of the mechanisms by which apoptosis may be initiated in a cell [164]. Increased apoptosis in immune cells has been reported for ME/CFS and Long COVID [165,233].

### 15.8. Dysbiosis

Dysfunctional autophagy may contribute to dysbiosis, reported to occur in ME/CFS [99] and Long COVID [234]. Where autophagy fails (e.g., in intestinal epithelial cells), it leads to ineffective pathogen removal (dysfunctional xenophagy, see above), reduced antimicrobial peptides (AMPs) from Paneth cells [235], and impaired intestinal barrier integrity (“leaky gut”) [236]. This would further produce endotoxins, such as lipopolysaccharides (LPS) and harmful metabolites [237], which can inhibit autophagy [170], allowing toxic protein aggregates to accumulate.

### 15.9. Dysautonomia

Dysautonomia, believed to promote glymphatic failure [238], is widely recognized as a prominent feature of ME/CFS [239] and Long COVID [240], potentially contributing to a number of key symptoms, including postural orthostatic tachycardia syndrome (POTS) and orthostatic hypotension, as part of orthostatic intolerance (OI) [241]. Misfolded proteins are believed to cause dysautonomia by accumulating in the peripheral nerves [242]. Accordingly, a dysfunctional autophagy can contribute to the development of dysautonomia.

Vagus nerve stimulation, which is believed to improve symptoms in ME/CFS [243] and Long COVID [244], can potentially stimulate autophagy [245], and restore autonomic function [246]. There are a number of clinical trials for vagus nerve stimulation [247].

### 15.10. Lactic Acidosis

Lactate has been reported to be elevated in ME/CFS and Long COVID, correlated with the severity of PEM [248]. It functions as a signaling molecule that can regulate autophagy [249]. Also, when misfolded proteins accumulate in mitochondria, mitophagy is triggered to clear the related damage, the failure of which will force the cells to rely on glycolysis and an overproduction of lactate, resulting in lactic acidosis.

### 15.11. Decreased Cerebral Blood Flow

The role of misfolded protein aggregates in causing decreased cerebral blood flow has been extensively investigated for Alzheimer’s disease related to the amyloid-β (Aβ) accumulation (e.g., ref. [250]). These structures are typically larger than the smallest capillaries, with the possibility of causing blockages that may hinder red blood cell flow. As for ME/CFS [5,251] and Long COVID [6,252], such reduction in blood flow may potentially be caused by the formation of the fibrinaloid microclots known to occur in these conditions [18,19,20], which may remain in blood circulation under a dysfunctional autophagy. Additionally, the reduction in red blood cell deformability reported for ME/CFS and Long COVID [253,254,255,256,257] could exacerbate the situation.

### 15.12. Olfactory Dysfunction

Olfactory dysfunction (OD) is a common complication in various neurologic and neurodegenerative disorders [258], with loss of olfactory function being one of the initial symptoms, and a potential biomarker of neurodegeneration [259]. OD in ME/CFS and Long COVID is believed to be interconnected post-viral phenomena. While ME/CFS patients sometimes experience a heightened sensitivity to smells, those with Long COVID predominantly endure persistent loss or distortion of smell [260]. Moreover, nearly 85% of Long COVID patients have been reported to experience some degree of OD [261], with the possibility of congestion of the glymphatic system, occurring as a result of damage to the olfactory sensory neurons [262].

One of the proposed mechanisms related to the present discussion is the amyloidogenesis of odorant-binding proteins (OBPs) [263]. It has been suggested that the high cytotoxicity of these amyloid aggregates may involve degradation of receptor neurons and the olfactory epithelial cells, and result in complete loss of function of OBPs [263].

## 16. Specific Regulators of Autophagy as Potential Therapeutic Targets for Its Modulation

It has been reported that the autophagy–lysosomal system is impaired in some neurodegenerative diseases [264], with the amyloid plaques remaining undegraded, causing neuronal death. Dysregulation of autophagy has also been associated with a number of other diseases, including various infections, inflammation, metabolic dysfunction and cancer.

**Figure 11:**
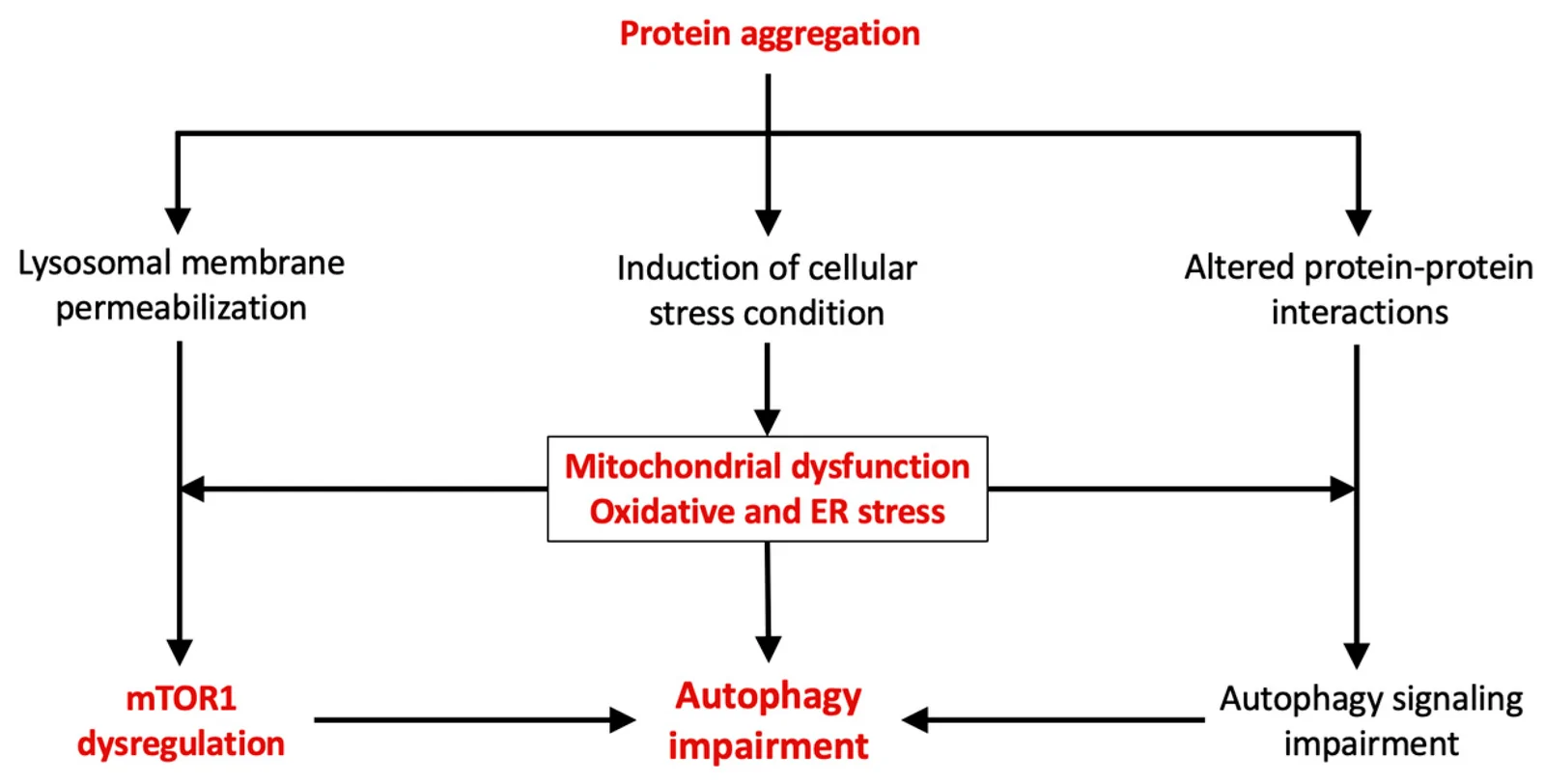
The proposed mechanisms by which the accumulation of protein aggregates may interfere with normal cellular/signaling pathways leading to autophagy impairment in various human diseases, including ME/CFS and Long COVID. The relationship with mTOR1 is indicated as a typical regulator of autophagy.

Evidence suggests that autophagy may be impaired in ME/CFS [17,64] and Long COVID [265]. Studies have shown elevated levels of the autophagy-related protein ATG13 in the serum of ME/CFS patients, indicating a potential disruption in the autophagy process [17,64]. This impairment may be linked to the chronic activation of mTOR, a protein kinase involved in cellular metabolism, and could contribute to cellular stress, immune activation, and reduced energy production (Figure 11) [64]. Some of the established regulators of autophagy are presented below, with specific discussions related to ME/CFS and Long COVID, and potential therapeutic strategies have been included. Some regulators of autophagy are next presented together with discussion of their possible relevance to ME/CFS and Long COVID. The regulators discussed here differ considerably in their evidentiary support as therapeutic targets for these conditions specifically, and none currently constitute an established treatment.

In a recent report describing enhancing autophagy in COVID-19, the use of a number of natural products, some of which (e.g., resveratrol and curcumin) have been previously shown to prevent amyloid aggregation [266,267,268,269], have been described [270].

### 16.1. mTOR1

mTOR1 (mechanistic target of rapamycin) is widely recognized as a master negative regulator of autophagy [271]. Under nutrient-rich conditions, it is active, and as a serine/threonine protein kinase, phosphorylates ATG13 (an autophagy-related protein) and ULK1 (unc-51-like kinase 1), thereby preventing autophagy [271]. When nutrient levels decline, mTORC1 is inactivated, leading to dephosphorylation of ATG13 and ULK1, allowing autophagy to proceed. Both ATG13 and ULK1 are involved in autophagosome formation, the defining hallmark of autophagy.

mTOR1 is recruited to the lysosomal membrane for activation in response to nutrients, and under nutrition deficiency is released from the lysosomal surface and becomes inactivated [272]. The structural and functional integrity of the lysosomal membrane is therefore critical for mTOR1 binding. mTOR1 is now recognized as a potential therapeutic target for modulating autophagy and has been recently discussed for ME/CFS and Long COVID [64,273]. The ME/CFS study [64] has been described as the first clinical trial involving mTOR activation and subsequent improvement in autophagy flux in reduction of PEM. Some of the limitations of the study have been pointed out by the authors [64].

### 16.2. Transcription Factor EB (TFEB)

Transcription factor EB (TFEB) is a master transcriptional regulator of autophagy and lysosomal biogenesis [274], and controls gene expression through binding to the CLEAR (Coordinated Lysosomal Expression and Regulation) network, which is a therapeutic target [275]. Thus, TFEB plays a key role in the degradation and clearance of protein aggregates [276]. Emerging research indicates that TFEB is downregulated or functionally suppressed in Long COVID, resulting in the impairment of lysosomal function and autophagy, and providing a potential therapeutic strategy for this condition [265].

### 16.3. AMP-Activated Protein Kinase (AMPK)

AMPK is a key regulator of metabolism and autophagy [277]. It acts as a primary energy sensor inside cells, detecting drops in ATP levels, promoting autophagy during energy starvation. When energy is low, AMP kinase phosphorylates specific serine/threonine residues on the ULK1 kinase complex (Unc-51-like kinase 1) causing its activation. The activation of ULK1 results in the suppression of mTOR1, allowing autophagy to proceed. Accordingly, AMPK has been described as a potential therapeutic target for modulating autophagy in general, and more specifically for ME/CFS and Long COVID [278,279].

The pharmacological activation of AMPK has been demonstrated in cultured skeletal muscle cells from ME/CFS patients [278], and AMPK has been independently proposed as a therapeutic target for Long COVID complications [279].

### 16.4. Beclin 1 Complex

Beclin 1 complex is widely recognized as an important regulator of autophagy [280] and plays a critical role in linking autophagy and apoptosis [281], with the latter being a key driver of ME/CFS [165] and Long COVID [233]. It is a master regulator of the Class III phosphatidylinositol 3-kinase (PI3KC3) complex, which is involved in early stages of autophagosome formation, and may serve as a therapeutic target for modulating autophagy [282].

Although no specific report seems to have been made related to its role in the regulation of Beclin-1 in ME/CFS or Long COVID, its association with disease severity in COVID-19 has been described [283].

### 16.5. Unc-51-like Kinase (ULK1) Complex

ULK1, together with ATG13, FIP200, and ATG101, forms the ULK1 initiation complex that carries out the first committed step of autophagosome formation [284]. ULK1 activity is directly controlled by the nutrient-sensing protein kinases mTORC1 and AMPK and has been proposed as a druggable node for pharmacologically tuning autophagy [285]. In a pilot study of ME/CFS patients, it was concluded that ULK1 expression may track with autophagy impairment in ME/CFS and could represent a modulatable target [286].

### 16.6. PTEN-Inducible Kinase 1 (PINK1)/Parkin Pathway

PINK1 and Parkin constitute a ubiquitin-dependent surveillance and quality control pathway that selectively targets damaged mitochondria [287] and has been investigated as a therapeutic target in a number of studies related to mitophagy (e.g., ref. [288]). As discussed in Section 7.2 above, mitophagy is a critical process in ME/CFS and Long COVID.

### 16.7. Sirtuin 1 (SIRT1)

SIRT1 is an NAD+-dependent deacetylase that promotes autophagy by deacetylating (causing activation of) multiple ATG proteins and has been suggested of possible value related to the management of COVID-19 [289]. Moreover, NAD+ depletion and reduction in SIRT1 activity have been proposed as contributors to the persistent oxidative stress and immune dysregulation described in Long COVID and ME/CFS [107,290], positioning SIRT1 restoration as a candidate strategy for normalizing autophagy in these conditions.

### 16.8. Nuclear Factor Erythroid 2-Related Factor 2 (NRF2)

As the master transcriptional regulator of the cellular antioxidant response, NRF2 directly upregulates the genes involved in autophagosome formation [291]. Reduced NRF2-dependent antioxidant capacity has been proposed to contribute to oxidative stress in lymphocytes from both ME/CFS and Long COVID patients, providing a direct rationale for NRF2-targeted modulation of autophagy in these conditions [107].

## 17. Summary and Concluding Remarks

ME/CFS and Long COVID are complex multisystem disorders and share similar biological mechanisms, including neuroimmune dysregulation, with no effective treatments. In recent years, the possibility of glymphatic and autophagy impairments have been suggested as parts of their pathophysiology [10,11,12,13,17,64], which have been discussed in the present perspective. We have tried to explain how the two mechanisms may work together to prevent the accumulation of misfolded proteins which could otherwise cause pathology as indicated below.

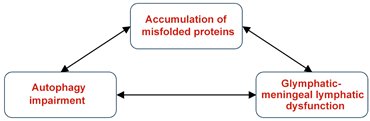
**Sources of misfolded proteins** Partially unfolded intermediates Cell debris Viral infection Oxidative stress/lipid peroxidation Gut dysbiosis Axonal damage Metabolic dysregulation**Types of autophagy** Aggrephagy Mitophagy ER-phagy Xenophagy Pexophagy Ferritinophagy Lipophagy Glycophagy**Regulators of autophagy** mTOR1 TFEB AMPK Beclin 1 complex ULK1 complex PINK1/Parkin pathway SIRT1 NRF2**Core pathophysiologies** Oxidative stress ER stress (neuro)inflammation) Mitochondrial dysfunction Endothelial dysfunction BBB dysfunction Decreased (cerebral) blood flow Mast cell activation T cell exhaustion**Some of the main symptoms** Fatigue PEM Sleep dysfunction Pain Brain fog Dysautonomia and POTS Olfactory dysfunction**Schematic and table** describing the relationship between misfolded protein accumulation, autophagy impairment and glymphatic–meningeal lymphatic dysfunction in ME/CFS and Long COVID. Some of the main sources of misfolded proteins, autophagy subtypes, regulators of autophagy, core pathophysiologies and symptoms are included.

Some of the potential sources of misfolded protein aggregates, different types of autophagy, and their regulators have been described. Of special note is the occurrence of amyloid microclots in the plasma samples of ME/CFS and Long COVID patients [18,19,20], resembling prions [22], further highlighting the significance of autophagy dysregulation.

Amongst the regulators of autophagy, mTORC1 appears to be of most direct relevance to ME/CFS and Long COVID. Low-dose rapamycin, an mTOR1 inhibitor, has been shown to alleviate fatigue and post-exertional malaise in ME/CFS patients via the improvement of autophagy [64], and its dysregulation has been proposed as a unifying mechanism for ME/CFS, Long COVID and related post-infectious syndromes [273].

Possible significance of an intimate cooperation between the glymphatic–meningeal lymphatic system and autophagy for maintaining tissue homeostasis in the CNS has been discussed. This is based on the premise that the biological functions of the two systems are complementary; one providing a mechanism of “mechanical” flushing out of waste products between the cells, and an autophagy–lysosomal pathway, which causes their enzymatic degradation inside the cells to small non-toxic components for recycling. Accordingly, we argue this interconnected waste removal network potentially plays an important role toward completion of the waste removal process in the brain, for maintaining homeostasis—ideas that require validation. Our primary focus involves misfolded protein aggregates, their production and propagation under different unfavorable situations, and how they may influence function. By describing different types of autophagy, we have provided a comprehensive account of the specific mechanistic features of this protein degradation mechanism and have described the related unfavorable consequences of their dysfunction. Furthermore, we have outlined some of the main mechanistic features of misfolded protein toxicity, such as membrane permeabilization, as previously reported by us and many other investigators related to neurodegenerative diseases.

Autophagy dysfunction in specific cell types, including microglia, neurons, astrocytes and T cells, has been described, and the consequences of the accumulation of misfolded proteins occurring as a result have been proposed (see Section 8), providing new insights into impaired protein degradation in these cells. One important conclusion of studies related to the potential mechanism of toxicity in neurological diseases has been the idea that misfolded aggregates that develop from disease-related and disease-unrelated proteins may behave similarly by a “detergent-like” mechanism due to their amphipathic characteristics.

ME/CFS and Long COVID are heterogeneous syndromes, comprising multiple biological subtypes. Accordingly, the proposed glymphatic–autophagy–proteostasis network described above may apply more strongly to particular patient phenotypes than to the conditions as a whole. Potential sources of this heterogeneity include differences in disease onset and duration, neuroimmune phenotypes, severity of sleep abnormalities, degree and type of autonomic dysfunction, metabolic status, and inflammatory profile. Future studies that more clearly establish a better understanding of the pathophysiology of these conditions would be needed to determine which subgroups most clearly exhibit the mechanistic features, and to guide more personalized therapeutic strategies.

Potential modulators of glymphatic dysfunction and autophagy impairment have been separately suggested by us and others in previous communications for ME/CFS [11,64]. Based on the discussions presented in this perspective, we would like to propose that a “holistic” strategy, involving the use of a cocktail of ingredients simultaneously addressing possible impairments in both the mechanical and biochemical components of the network discussed here, may prove more effective. Finally, we hope the present perspective will guide future research to better understand the neurological aspects of ME/CFS and Long COVID.

## Data Availability

No new data were created or analyzed in this study. Data sharing is not applicable to this article.

## References

[1] Annesley S.J., Missailidis D., Heng B., Josev E.K., Armstrong C.W. (2024). Unravelling shared mechanisms: Insights from recent ME/CFS research to illuminate long COVID pathologies. Trends Mol. Med..

[2] Faghy M.A., Wüst R.C.I., Altmann D.M., Ashton R.E., McMullen S.B., Duncan R., Ewing A.G., Hausmann E., Gupta S., Hornig M. (2026). Current status and future perspectives on the mechanistic and pathophysiological understanding of long COVID. Commun. Med..

[3] Komaroff A.L., Dantzer R. (2025). Causes of symptoms and symptom persistence in long COVID and myalgic encephalomyelitis/chronic fatigue syndrome. Cell Rep. Med..

[4] VanElzakker M.B., Bues H.F., Brusaferri L., Kim M., Saadi D., Ratai E.-M., Dougherty D.D., Loggia M.L. (2024). Neuroinflammation in post-acute sequelae of COVID-19 (PASC) as assessed by [11C] PBR28 PET correlates with vascular disease measures. Brain Behav. Immun..

[5] Biswal B., Kunwar P., Natelson B.H. (2011). Cerebral blood flow is reduced in chronic fatigue syndrome as assessed by arterial spin labeling. J. Neurol. Sci..

[6] Kutz D.F., Garbsch R., Mooren F.C., Schmitz B., Voelcker-Rehage C. (2026). Assessment of dynamic cerebral blood flow changes during cognitive tasks in patients with post-COVID-19 syndrome. Brain Commun..

[7] Jensen M.A., Dafoe M.L., Wilhelmy J., Cervantes L., Okumu A.N., Kipp L., Nemat-Gorgani M., Davis R.W. (2023). Catalytic antibodies may contribute to demyelination in Myalgic Encephalomyelitis/Chronic Fatigue Syndrome. Biochemistry.

[8] Khodanovich M., Kamaeva D., Usova A., Pashkevich V., Moshkina M., Obukhovskaya V., Kataeva N., Levina A., Tumentceva Y., Shadrina M. (2025). Demyelination and Cognitive Performance in Long COVID Patients with Insomnia and/or Depression. Int. J. Mol. Sci..

[9] Frid K., Einstein O., Friedman-Levi Y., Binyamin O., Ben-Hur T., Gabizon R. (2015). Aggregation of MBP in chronic demyelination. Ann. Clin. Transl. Neurol..

[10] Wostyn P., De Deyn P.P. (2018). The putative glymphatic signature of chronic fatigue syndrome: A new view on the disease pathogenesis and therapy. Med. Hypotheses.

[11] Nemat-Gorgani M., Jensen M.A., Davis R.W. (2025). Glymphatic System Dysregulation as a Key Contributor to Myalgic Encephalomyelitis/Chronic Fatigue Syndrome. Int. J. Mol. Sci..

[12] Wu L., Zhang Z., Liang X., Wang Y., Cao Y., Li M., Zhou F. (2024). Glymphatic system dysfunction in recovered patients with mild COVID-19: A DTI-ALPS study. Iscience.

[13] Thapaliya K., Marshall-Gradisnik S., Inderyas M., Barnden L. (2026). Disrupted glymphatic function and its relationship with sleep and cognitive impairment in ME/CFS assessed via DTI-ALPS. Front. Neurosci..

[14] Chaganti J.R., Talekar T.K., Brew B.J. (2025). Asymmetrical glymphatic dysfunction in patients with long Covid associated neurocognitive impairment-correlation with BBB disruption. BMC Neurol..

[15] He C., Xie J., Fang W., Guo B., Shi Y., Li A., Liu H., Zhu Z., Bao W., Niu X. (2025). Dynamic brain glymphatic changes and cognitive function in COVID-19 recovered patients: A DTI-ALPS prospective cohort study. Front. Psychol..

[16] Tang Y.-L., Chen H.-B., Liu P., Liao Y.-H., Xie A. (2025). Glymphatic function alterations in sleep disorder patients post-COVID-19: A longitudinal DTI-ALPS study. Nat. Sci. Sleep.

[17] Gottschalk G., Peterson D., Knox K., Maynard M., Whelan R.J., Roy A. (2022). Elevated ATG13 in serum of patients with ME/CFS stimulates oxidative stress response in microglial cells via activation of receptor for advanced glycation end products (RAGE). Mol. Cell. Neurosci..

[18] Nunes J.M., Kruger A., Proal A., Kell D.B., Pretorius E. (2022). The occurrence of hyperactivated platelets and fibrinaloid microclots in myalgic encephalomyelitis/chronic fatigue syndrome (ME/CFS). Pharmaceuticals.

[19] Kell D.B., Khan M.A., Kane B., Lip G.Y.H., Pretorius E. (2024). Possible role of fibrinaloid microclots in Postural Orthostatic Tachycardia Syndrome (POTS): Focus on Long COVID. J. Pers. Med..

[20] Nunes M., Kell L., Slaghekke A., Wüst R.C., Fielding B.C., Kell D.B., Pretorius E. (2026). Virus-induced endothelial senescence as a cause and driving factor for ME/CFS and long COVID: Mediated by a dysfunctional immune system. Cell Death Dis..

[21] Kell D.B., Laubscher G.J., Pretorius E. (2022). A central role for amyloid fibrin microclots in long COVID/PASC: Origins and therapeutic implications. Biochem. J..

[22] Kell D.B., Pretorius E. (2023). Are fibrinaloid microclots a cause of autoimmunity in Long Covid and other post-infection diseases?. Biochem. J..

[23] Tiwari D., Singh V.K., Baral B., Pathak D.K., Jayabalan J., Kumar R., Tapryal S., Jha H.C. (2021). Indication of neurodegenerative cascade initiation by amyloid-like aggregate-forming EBV proteins and peptide in Alzheimer’s disease. ACS Chem. Neurosci..

[24] Nystrom S., Hammarstrom P. (2022). Amyloidogenesis of SARS-CoV-2 spike protein. J. Am. Chem. Soc..

[25] Palmer J.E., Wilson N., Son S.M., Obrocki P., Wrobel L., Rob M., Takla M., Korolchuk V.I., Rubinsztein D.C. (2025). Autophagy, aging, and age-related neurodegeneration. Neuron.

[26] Park E.-J., Jo E.-K., Paik S. (2025). Overview of the autophagy pathways associated with infection. J. Bacteriol. Virol..

[27] Lyu J., Zhang H., Wang C., Pan M. (2024). New insight in treating autoimmune diseases by targeting autophagy. Autoimmunity.

[28] Spicer C.M., Chu B.X., Volberding P.A. (2024). Chronic Conditions Similar to Long COVID. Long-Term Health Effects of COVID-19: Disability and Function Following SARS-CoV-2 Infection.

[29] Murovska M., Krumina A., Araja D., Kujawski S., Zalewski P., Nora-Krukle Z., Berkis U. (2026). Myalgic encephalomyelitis/chronic fatigue syndrome and fibromyalgia-overlap, differences, and emerging insights. J. Transl. Med..

[30] Licastro E., Pignataro G., Iliff J.J., Xiang Y., Lo E.H., Hayakawa K., Esposito E. (2024). Glymphatic and lymphatic communication with systemic responses during physiological and pathological conditions in the central nervous system. Commun. Biol..

[31] Kim M.W., Kipnis J. (2025). Glymphatics and meningeal lymphatics unlock the brain-immune code. Immunity.

[32] Wang H., Wang X., Liu N., Wang H., Feng C. (2026). Glymphatic–meningeal lymphatic system imbalance: A peripheral-to-central inflammatory bridge in perioperative neurocognitive disorders. Front. Immunol..

[33] Sun H., Fan H., Zhou Y., Zhu H., Chen Y., Zhang R., Wang K., Pan Y., Zhang A. (2026). The glymphatic system and meningeal lymphatics: Current understandings and future perspectives. MedComm.

[34] Raper D., Louveau A., Kipnis J. (2016). How do meningeal lymphatic vessels drain the CNS?. Trends Neurosci..

[35] Iliff J.J., Wang M., Liao Y., Plogg B.A., Peng W., Gundersen G.A., Benveniste H., Vates G.E., Deane R., Goldman S.A. (2012). A paravascular pathway facilitates CSF flow through the brain parenchyma and the clearance of interstitial solutes, including amyloid β. Sci. Transl. Med..

[36] Proulx S.T. (2021). Cerebrospinal fluid outflow: A review of the historical and contemporary evidence for arachnoid villi, perineural routes, and dural lymphatics. Cell Mol. Life Sci..

[37] Vinje V., Eklund A., Mardal K.-A., Rognes M.E., Støverud K.-H. (2020). Intracranial pressure elevation alters CSF clearance pathways. Fluids Barriers CNS.

[38] Sokołowski W., Barszcz K., Kupczyńska M., Czubaj N., Skibniewski M., Purzyc H. (2018). Lymphatic drainage of cerebrospinal fluid in mammals–are arachnoid granulations the main route of cerebrospinal fluid outflow?. Biologia.

[39] Smyth L.C.D., Xu D., Okar S.V., Dykstra T., Rustenhoven J., Papadopoulos Z., Bhasiin K., Kim M.W., Drieu A., Mamuladze T. (2024). Identification of direct connections between the dura and the brain. Nature.

[40] Louveau A., Smirnov I., Keyes T.J., Eccles J.D., Rouhani S.J., Peske J.D., Derecki N.C., Castle D., Mandell J.W., Lee K.S. (2015). Structural and functional features of central nervous system lymphatic vessels. Nature.

[41] Aspelund A., Antila S., Proulx S.T., Karlsen T.V., Karaman S., Detmar M., Wiig H., Alitalo K. (2015). A dural lymphatic vascular system that drains brain interstitial fluid and macromolecules. J. Exp. Med..

[42] Ciechanover A., Kwon Y.T. (2017). Protein quality control by molecular chaperones in neurodegeneration. Front. Neurosci..

[43] Pohl C., Dikic I. (2019). Cellular quality control by the ubiquitin-proteasome system and autophagy. Science.

[44] Lei L., Wu Z., Winklhofer K.F. (2021). Protein quality control by the proteasome and autophagy: A regulatory role of ubiquitin and liquid-liquid phase separation. Matrix Biol..

[45] Bayer T.A. (2015). Proteinopathies, a core concept for understanding and ultimately treating degenerative disorders?. Eur. Neuropsychopharmacol..

[46] Borlepawar A., Neu M., Ma Z., Deshpande A., Bühringer H., Hajieva P., Frey N., Rangrez A.Y. (2026). Emerging perspectives in proteostasis: Bridging mechanisms and therapeutics for human diseases. Signal Transduct. Target. Ther..

[47] Funderburk S.F., Marcellino B.K., Yue Z. (2010). Cell “self-eating” (autophagy) mechanism in Alzheimer’s disease. Mt. Sinai J. Med..

[48] Wang H.-D., Lv C.-L., Feng L., Guo J.-X., Zhao S.-Y., Jiang P. (2024). The role of autophagy in brain health and disease: Insights into exosome and autophagy interactions. Heliyon.

[49] De Duve C. (1963). The lysosome. Sci. Am..

[50] Braun M., Simon M.J., Jang J., Sanderson K., Swierz J., Sevao M., Pincus A.B., Schaser A.J., Elliott J.E., Lim M.M. (2024). Aquaporin-4 mis-localization slows glymphatic clearance of α-synuclein and promotes α-synuclein pathology and aggregate propagation. bioRxiv.

[51] Wang X., Xu Z., Cai Y., Zeng S., Peng B., Ren X., Yan Y., Gong Z. (2020). Rheostatic balance of circadian rhythm and autophagy in metabolism and disease. Front. Cell Dev. Biol..

[52] McKee C.A., Polino A.J., King M.W., Musiek E.S. (2023). Circadian clock protein BMAL1 broadly influences autophagy and endolysosomal function in astrocytes. Proc. Natl. Acad. Sci. USA.

[53] Saurel M., Fornasieri I., Del Sordo G.C., Chatain C., Fantini M.L., Gruet M., Saidi O. (2026). Sleep in myalgic encephalomyelitis/chronic fatigue syndrome shows marked night-to-night fluctuation under free-living conditions—Results from a matched case-control study. J. Clin. Sleep Med..

[54] Maksoud R., Eaton-Fitch N., Matula M., Cabanas H., Staines D., Marshall-Gradisnik S. (2021). Systematic review of sleep characteristics in myalgic encephalomyelitis/chronic fatigue syndrome. Healthcare.

[55] Wilson A., Mejia G.C.R., Lomba S., Geng L.N., Malunjkar S., Bonilla H., Sum-Ping O. (2025). A Multidimensional Assessment of Sleep Disorders in Long COVID Using the Alliance Sleep Questionnaire. Healthcare.

[56] Livieratos A., Lockley S.W., Tsiodras S. (2025). Post infectious fatigue and circadian rhythm disruption in long-COVID and other infections: A need for further research. EClinicalMedicine.

[57] Reddy O.C., van der Werf Y.D. (2020). The sleeping brain: Harnessing the power of the glymphatic system through lifestyle choices. Brain Sci..

[58] Das N., Dhamija R., Sarkar S. (2024). The role of astrocytes in the glymphatic network: A narrative review. Metab. Brain Dis..

[59] Mohamed A.Z., Andersen T., Radovic S., Del Fante P., Kwiatek R., Calhoun V., Bhuta S., Hermens D.F., Lagopoulos J., Shan Z.Y. (2023). Objective sleep measures in chronic fatigue syndrome patients: A systematic review and meta-analysis. Sleep Med. Rev..

[60] Jackson M.L., Bruck D. (2012). Sleep abnormalities in chronic fatigue syndrome/myalgic encephalomyelitis: A review. J. Clin. Sleep Med..

[61] Tedjasukmana R., Budikayanti A., Islamiyah W.R., Witjaksono A.M.A.L., Hakim M. (2023). Sleep disturbance in post COVID-19 conditions: Prevalence and quality of life. Front. Neurol..

[62] Ibrahim A., Cesari M., Tang Q., Aktan Süzgün M., Brandauer E., Holzknecht E., Wachter A., Anselmi V., Heidbreder A., Stefani A. (2026). Sleep architecture and rapid eye movement sleep without atonia in post-COVID-19 insomnia. Sleep.

[63] Szypulski K., Tyszka A., Pyza E., Damulewicz M. (2024). Autophagy as a new player in the regulation of clock neurons physiology of Drosophila melanogaster. Sci. Rep..

[64] Ruan B.T., Bulbule S., Gile B., Reyes A., Chheda B., Bateman L., Bell J., Yellman B., Grach S.L., Berner J. (2025). Low-dose rapamycin alleviates clinical symptoms of fatigue and PEM in ME/CFS patients via improvement of autophagy: A pilot study. J. Transl. Med..

[65] Kell D.B., Khan M.A., Pretorius E. (2024). Fibrinaloid microclots in long COVID: Assessing the actual evidence properly. Res. Pract. Thromb. Haemost..

[66] Tate W., Walker M., Sweetman E., Helliwell A., Peppercorn K., Edgar C., Blair A., Chatterjee A. (2022). Molecular mechanisms of neuroinflammation in ME/CFS and long COVID to sustain disease and promote relapses. Front. Neurol..

[67] Lee J.-S., Sato W., Son C.-G. (2024). Brain-regional characteristics and neuroinflammation in ME/CFS patients from neuroimaging: A systematic review and meta-analysis. Autoimmun. Rev..

[68] Glassford J.A. (2017). The neuroinflammatory etiopathology of myalgic encephalomyelitis/chronic fatigue syndrome (ME/CFS). Front. Physiol..

[69] Kuzu O.F., Granerud L.J.T., Saatcioglu F. (2025). Navigating the landscape of protein folding and proteostasis: From molecular chaperones to therapeutic innovations. Signal Transduct. Target. Ther..

[70] Thambirajah A.A., Sleigh K., Stiver H.G., Chow A.W., Aw C. (2008). Differential heat shock protein responses to strenuous standardized exercise in chronic fatigue syndrome patients and matched healthy controls. Clin. Investig. Med..

[71] Mitra R., Wu K., Lee C., Bardwell J.C. (2022). ATP-independent chaperones. Annu. Rev. Biophys..

[72] Syed A.M., Karius A.K., Ma J., Wang P.-Y., Hwang P.M. (2025). Mitochondrial dysfunction in myalgic encephalomyelitis/chronic fatigue syndrome. Physiology.

[73] Macnaughtan J., Chau K.-Y., Brennan E., Toffoli M., Spinazzola A., Hillman T., Heightman M., Schapira A.H.V. (2025). Mitochondrial function is impaired in long COVID patients. Ann. Med..

[74] Rutherford G., Manning P., Newton J.L. (2016). Understanding muscle dysfunction in chronic fatigue syndrome. J. Aging Res..

[75] Reyburn-Orne T., Anderson M. (2026). Pain and Long COVID Rehab: A Conflict of Paradigms?. Pain Manag. Nurs..

[76] Jahan F., Nanji K., Qidwai W., Qasim R. (2012). Fibromyalgia syndrome: An overview of pathophysiology, diagnosis and management. Oman Med. J..

[77] Haider S., Janowski A.J., Lesnak J.B., Hayashi K., Dailey D.L., Chimenti R., Frey-Law L.A., Sluka K.A., Berardi G. (2023). A comparison of pain, fatigue, and function between post–COVID-19 condition, fibromyalgia, and chronic fatigue syndrome: A survey study. Pain.

[78] Zhu F.-Q., Zhang W.-J., Guan B.-Z. (2025). The role of autophagy in chronic pain. J. Neuroimmunol..

[79] Fu L., Yang Y., Ma Y., Wang Y., Tang P., Liu J. (2026). Autophagy in neuropathic pain: Cell specificity, molecular mechanisms, and therapeutic prospects. J. Holist. Integr. Pharm..

[80] Myers R.R., Campana W.M., Shubayev V.I. (2006). The role of neuroinflammation in neuropathic pain: Mechanisms and therapeutic targets. Drug Discov. Today.

[81] Wijayanti I.A.S., Adnyana I.M.O., Widyadharma I.P.E., Wiratnaya I.G.E., Mahadewa T.G.B., Astawa I.N.M. (2024). Neuroinflammation mechanism underlying neuropathic pain: The role of mesenchymal stem cell in neuroglia. AIMS Neurosci..

[82] Ji R.-R., Xu Z.-Z., Gao Y.-J. (2014). Emerging targets in neuroinflammation-driven chronic pain. Nat. Rev. Drug Discov..

[83] Echeverria-Villalobos M., Tortorici V., Brito B.E., Ryskamp D., Uribe A., Weaver T. (2023). The role of neuroinflammation in the transition of acute to chronic pain and the opioid-induced hyperalgesia and tolerance. Front. Pharmacol..

[84] Findeisen K., Guymer E., Littlejohn G. (2025). Neuroinflammatory and immunological aspects of fibromyalgia. Brain Sci..

[85] González-Álvarez M.E., Riquelme-Aguado V., González-Pérez Á., Murillo-Llergo R., Manjón-Olmedillas M., Turroni S., Rossettini G., Villafañe J.H. (2024). Association Between Systemic Neuroinflammation, Pain Perception and Clinical Status in Fibromyalgia Patients: Cross-Sectional Study. Cells.

[86] Uversky V.N. (2008). Amyloidogenesis of natively unfolded proteins. Curr. Alzheimer Res..

[87] Abedini A., Cao P., Raleigh D.P. (2016). Detection of helical intermediates during amyloid formation by intrinsically disordered polypeptides and proteins. Protein Amyloid Aggregation: Methods and Protocols.

[88] Kurauskas V., Tonelli M., Kirchdoerfer R.N., Henzler-Wildman K. (2025). The C-terminal domain of SARS-CoV-2 nsp8 is a molten globule in the absence of binding partners. J. Mol. Biol..

[89] Wang W.-H., Chang L.-K., Liu S.-T. (2011). Molecular interactions of Epstein-Barr virus capsid proteins. J. Virol..

[90] Lopes T.R.R., Sitton B.S., Pillat M.M., Mello C.F., Weiblen R., Flores E.F., Silva Júnior J.V.J. (2025). SARS-CoV-2 and neurotropism: Evidence, gaps and reflections. J. Med. Microbiol..

[91] Qin C., Chu Y.-H., Zhou L.-Q., Dong M.-H., Wang W., Tian D.-S. (2023). Haste makes waste: Staged suppression of autophagic-lysosomal pathway in microglia promotes the efficient clearance of myelin debris. Autophagy.

[92] Chagas L.d.S., Serfaty C.A. (2024). The influence of microglia on neuroplasticity and long-term cognitive sequelae in long COVID: Impacts on brain development and beyond. Int. J. Mol. Sci..

[93] Thapa R., Bhat A.A., Shahwan M., Ali H., PadmaPriya G., Bansal P., Rajotiya S., Barwal A., Prasad G.S., Pramanik A. (2024). Proteostasis disruption and senescence in Alzheimer’s disease pathways to neurodegeneration. Brain Res..

[94] Proal A., Marshall T. (2018). Myalgic Encephalomyelitis/Chronic Fatigue Syndrome in the Era of the Human Microbiome: Persistent Pathogens Drive Chronic Symptoms by Interfering With Host Metabolism, Gene Expression, and Immunity. Front. Pediatr..

[95] Wang P.-Y., Ma J., Kim Y.-C., Son A.Y., Syed A.M., Liu C., Mori M.P., Huffstutler R.D., Stolinski J.L., Talagala S.L. (2023). WASF3 disrupts mitochondrial respiration and may mediate exercise intolerance in myalgic encephalomyelitis/chronic fatigue syndrome. Proc. Natl. Acad. Sci. USA.

[96] Stefano G.B., Büttiker P., Weissenberger S., Anders M., Raboch J., Ptacek R., Kream R.M. (2023). Potential prion involvement in long COVID-19 neuropathology, including behavior. Cell. Mol. Neurobiol..

[97] Christ W., Kapell S., Sobkowiak M.J., Mermelekas G., Evertsson B., Sork H., Saher O., Bazaz S., Gustafsson O., Cardenas E.I. (2024). SARS-CoV-2 and HSV-1 induce amyloid aggregation in human CSF resulting in drastic soluble protein depletion. ACS Chem. Neurosci..

[98] Kowalski K., Mulak A. (2019). Brain-gut-microbiota axis in Alzheimer’s disease. J. Neurogastroenterol. Motil..

[99] König R.S., Albrich W.C., Kahlert C.R., Bahr L.S., Löber U., Vernazza P., Scheibenbogen C., Forslund S.K. (2022). The gut microbiome in myalgic encephalomyelitis (ME)/chronic fatigue syndrome (CFS). Front. Immunol..

[100] Zollner A., Meyer M., Jukic A., Adolph T., Tilg H. (2024). The intestine in acute and long COVID: Pathophysiological insights and key lessons. Yale J. Biol. Med..

[101] Friedland R.P., Chapman M.R. (2017). The role of microbial amyloid in neurodegeneration. PLoS Pathog..

[102] Jin J., Xu Z., Zhang L., Zhang C., Zhao X., Mao Y., Zhang H., Liang X., Wu J., Yang Y. (2023). Gut-derived β-amyloid: Likely a centerpiece of the gut–brain axis contributing to Alzheimer’s pathogenesis. Gut Microbes.

[103] Khodanovich M.Y., Kamaeva D.A., Naumova A.V. (2022). Role of Demyelination in the Persistence of Neurological and Mental Impairments after COVID-19. Int. J. Mol. Sci..

[104] David M.A., Tayebi M. (2014). Detection of protein aggregates in brain and cerebrospinal fluid derived from multiple sclerosis patients. Front. Neurol..

[105] Lassmann H., Van Horssen J. (2011). The molecular basis of neurodegeneration in multiple sclerosis. FEBS Lett..

[106] Shen D., Liu K., Wang H., Wang H. (2022). Autophagy modulation in multiple sclerosis and experimental autoimmune encephalomyelitis. Clin. Exp. Immunol..

[107] Shankar V., Wilhelmy J., Curtis E.J., Michael B., Cervantes L., Mallajosyula V., Davis R.W., Snyder M., Younis S., Robinson W.H. (2025). Oxidative stress is a shared characteristic of ME/CFS and Long COVID. Proc. Natl. Acad. Sci. USA.

[108] Liu L., Komatsu H., Murray I.V., Axelsen P.H. (2008). Promotion of amyloid β protein misfolding and fibrillogenesis by a lipid oxidation product. J. Mol. Biol..

[109] Michalska P., León R. (2020). When it comes to an end: Oxidative stress crosstalk with protein aggregation and neuroinflammation induce neurodegeneration. Antioxidants.

[110] Reed T.T. (2011). Lipid peroxidation and neurodegenerative disease. Free Radic. Biol. Med..

[111] Joshi G., Perluigi M., Sultana R., Agrippino R., Calabrese V., Butterfield D.A. (2006). In vivo protection of synaptosomes by ferulic acid ethyl ester (FAEE) from oxidative stress mediated by 2, 2-azobis (2-amidino-propane) dihydrochloride (AAPH) or Fe^2+^/H_2_O_2_: Insight into mechanisms of neuroprotection and relevance to oxidative stress-related neurodegenerative disorders. Neurochem. Int..

[112] Angelova P.R., Esteras N., Abramov A.Y. (2021). Mitochondria and lipid peroxidation in the mechanism of neurodegeneration: Finding ways for prevention. Med. Res. Rev..

[113] Sandvik M.K., Sørland K., Leirgul E., Rekeland I.G., Stavland C.S., Mella O., Fluge Ø. (2023). Endothelial dysfunction in ME/CFS patients. PLoS ONE.

[114] Wu X., Xiang M., Jing H., Wang C., Novakovic V.A., Shi J. (2024). Damage to endothelial barriers and its contribution to long COVID. Angiogenesis.

[115] van Campen C.M., Rowe P.C., Visser F.C. (2023). Worsening symptoms is associated with larger cerebral blood flow abnormalities during tilt-testing in Myalgic Encephalomyelitis/Chronic Fatigue Syndrome (ME/CFS). Medicina.

[116] Wang Y., Yang Z., Zheng X., Liang X., Wu L., Wu C., Dai J., Cao Y., Li M., Zhou F. (2025). Cerebral blood flow alterations and host genetic association in individuals with long COVID: A transcriptomic-neuroimaging study. J. Cereb. Blood Flow. Metab..

[117] Mameli E., Martello A., Caporali A. (2022). Autophagy at the interface of endothelial cell homeostasis and vascular disease. FEBS J..

[118] Li G.-H., Han F.-F., Kalafatis E., Kong Q.-P., Xiao W. (2025). Systems modeling reveals shared metabolic dysregulation and potential treatments in ME/CFS and long COVID. Int. J. Mol. Sci..

[119] Hu C., Lin M., Wang C., Zhang S. (2025). Current Understanding of Protein Aggregation in Neurodegenerative Diseases. Int. J. Mol. Sci..

[120] Bestebroer J., V’KOvski P., Mauthe M., Reggiori F. (2013). Hidden behind autophagy: The unconventional roles of ATG proteins. Traffic.

[121] Klionsky D.J., Emr S.D. (2000). Autophagy as a regulated pathway of cellular degradation. Science.

[122] Lamark T., Johansen T. (2012). Aggrephagy: Selective disposal of protein aggregates by macroautophagy. Int. J. Cell Biol..

[123] Coppola-Segovia V., Reggiori F. (2024). Molecular insights into aggrephagy: Their cellular functions in the context of neurodegenerative diseases. J. Mol. Biol..

[124] Ding W.-X., Yin X.-M. (2012). Mitophagy: Mechanisms, pathophysiological roles, and analysis. Biol. Chem..

[125] Molnar T., Lehoczki A., Fekete M., Varnai R., Zavori L., Erdo-Bonyar S., Simon D., Berki T., Csecsei P., Ezer E. (2024). Mitochondrial dysfunction in long COVID: Mechanisms, consequences, and potential therapeutic approaches. Geroscience.

[126] Kopeć K., Szleszkowski S., Koziorowski D., Szlufik S. (2023). Glymphatic system and mitochondrial dysfunction as two crucial players in pathophysiology of neurodegenerative disorders. Int. J. Mol. Sci..

[127] Hanna R.A., Quinsay M.N., Orogo A.M., Giang K., Rikka S., Gustafsson Å.B. (2012). Microtubule-associated protein 1 light chain 3 (LC3) interacts with Bnip3 protein to selectively remove endoplasmic reticulum and mitochondria via autophagy. J. Biol. Chem..

[128] Warrayat A., Ali A., Waked J., Tocci D., Speth R.C. (2025). Assessment of the therapeutic potential of salubrinal for ME/CFS and long-COVID. Trends Mol. Med..

[129] Mao K., Klionsky D.J. (2017). Xenophagy: A battlefield between host and microbe, and a possible avenue for cancer treatment. Autophagy.

[130] Liang S., Wu Y.-S., Li D.-Y., Tang J.-X., Liu H.-F. (2021). Autophagy in viral infection and pathogenesis. Front. Cell Dev. Biol..

[131] Mijaljica D., Klionsky D.J. (2020). Autophagy/virophagy: A “disposal strategy” to combat COVID-19. Autophagy.

[132] Manjithaya R., Nazarko T.Y., Farré J.-C., Subramani S. (2010). Molecular mechanism and physiological role of pexophagy. FEBS Lett..

[133] Sun K., Li C., Liao S., Yao X., Ouyang Y., Liu Y., Wang Z., Li Z., Yao F. (2022). Ferritinophagy, a form of autophagic ferroptosis: New insights into cancer treatment. Front. Pharmacol..

[134] Liu M.Z., Kong N., Zhang G.Y., Xu Q., Xu Y., Ke P., Liu C. (2022). The critical role of ferritinophagy in human disease. Front. Pharmacol..

[135] Ruscitti P., Ursini F., Shoenfeld Y. (2023). Ferritin and myalgic encephalomyelitis/chronic fatigue syndrome in post COVID-19, an unexpected facet of the hyperferritinemic syndrome?. J. Psychosom. Res..

[136] Yamamoto Y., Otsuka Y., Tokumasu K., Sunada N., Nakano Y., Honda H., Sakurada Y., Hasegawa T., Hagiya H., Otsuka F. (2023). Utility of serum ferritin for predicting myalgic encephalomyelitis/chronic fatigue syndrome in patients with long COVID. J. Clin. Med..

[137] Sun J., Huo C., Li Y., Li X., Xu J., Tian J., Xiao J., Zhang R., Han J., Zhou L. (2026). Pseudorabies virus triggers ferritinophagy-mediated ferroptosis and neuroinflammation in viral encephalitis. Proc. Natl. Acad. Sci. USA.

[138] Mueller C., Lin J.C., Sheriff S., Maudsley A.A., Younger J.W. (2020). Evidence of widespread metabolite abnormalities in Myalgic encephalomyelitis/chronic fatigue syndrome: Assessment with whole-brain magnetic resonance spectroscopy. Brain Imaging Behav..

[139] Kavanagh E. (2022). Long Covid brain fog: A neuroinflammation phenomenon?. Oxf. Open Immunol..

[140] Jarocki M., Turek K., Saczko J., Tarek M., Kulbacka J. (2024). Lipids associated with autophagy: Mechanisms and therapeutic targets. Cell Death Discov..

[141] Missailidis D., Armstrong C.W., Anderson D., Allan C.Y., Sanislav O., Smith P.K., Esmaili T., Creek D.J., Annesley S.J., Fisher P.R. (2026). Multi-omics identifies lipid accumulation in Myalgic Encephalomyelitis/Chronic Fatigue Syndrome cell lines: A case-control study. J. Transl. Med..

[142] Seitz T., Setz C., Rauch P., Schubert U., Hellerbrand C. (2023). Lipid accumulation in host cells promotes SARS-CoV-2 replication. Viruses.

[143] Delbridge L.M., Koutsifeli P., Fong S.P., Annandale M., Weeks K.L., Bell J.R., Mellor K.M. (2022). Glycophagy—The physiological perspective on a newly characterized glycogen-selective autophagy. Curr. Opin. Physiol..

[144] Mellor K., Koutsifeli P., Varma U., Curl C., Janssens J., Raaijmakers A., Mills R., Hudson J., Porrello E., Bell J. (2024). Glycophagy is an Effective Therapeutic Target to Remediate Diastolic Dysfunction in Diabetic Heart Disease. Heart Lung Circ..

[145] Guo H., Li Y., Wang S., Yang Y., Xu T., Zhao J., Wang J., Zuo W., Wang P., Zhao G. (2024). Dysfunction of astrocytic glycophagy exacerbates reperfusion injury in ischemic stroke. Redox Biol..

[146] Cai Q., Ganesan D. (2022). Regulation of neuronal autophagy and the implications in neurodegenerative diseases. Neurobiol. Dis..

[147] Zeng J., Indajang J., Pitt D., Lo C.H. (2025). Lysosomal acidification impairment in astrocyte-mediated neuroinflammation. J. Neuroinflamm..

[148] Potokar M., Jorgačevski J. (2025). Targeting autophagy in astrocytes: A potential for neurodegenerative disease intervention. Front. Cell. Neurosci..

[149] Giusti V., Kaur G., Giusto E., Civiero L. (2024). Brain clearance of protein aggregates: A close-up on astrocytes. Mol. Neurodegener..

[150] Smethurst P., Franklin H., Clarke B.E., Sidle K., Patani R. (2022). The role of astrocytes in prion-like mechanisms of neurodegeneration. Brain.

[151] Sun H., Mullington J.M., Thomas R.J. (2026). Post-infection sleep syndrome: Long COVID as an example. Sleep.

[152] Lu H.-J., Gao Y.-J. (2023). Astrocytes in chronic pain: Cellular and molecular mechanisms. Neurosci. Bull..

[153] Cheng T., Xu Z., Ma X. (2022). The role of astrocytes in neuropathic pain. Front. Mol. Neurosci..

[154] Guldner I.H., Wagner V.P., Moran-Losada P., Shi S.M., Golub S.W., Hevler J.F., Chen K., Meese B.T., Ghoochani A., Pulido E. (2026). Ageing promotes microglial accumulation of slow-degrading synaptic proteins. Nature.

[155] Choi I., Wang M., Yoo S., Xu P., Seegobin S.P., Li X., Han X., Wang Q., Peng J., Zhang B. (2023). Autophagy enables microglia to engage amyloid plaques and prevents microglial senescence. Nat. Cell Biol..

[156] Jessen N.A., Munk A.S.F., Lundgaard I., Nedergaard M. (2015). The glymphatic system: A beginner’s guide. Neurochem. Res..

[157] Zou K., Deng Q., Zhang H., Huang C. (2023). Glymphatic system: A gateway for neuroinflammation. Neural Regen. Res..

[158] Ohgidani M., Kato T.A., Hosoi M., Tsuda M., Hayakawa K., Hayaki C., Iwaki R., Sagata N., Hashimoto R., Inoue K. (2017). Fibromyalgia and microglial TNF-α: Translational research using human blood induced microglia-like cells. Sci. Rep..

[159] Wang Y., Ma A., Song N.-J., Shannon A.E., Amankwah Y.S., Chen X., Wu W., Wang Z., Saadey A.A., Yousif A. (2025). Proteotoxic stress response drives T cell exhaustion and immune evasion. Nature.

[160] Iu D.S., Maya J., Vu L.T., Fogarty E.A., McNairn A.J., Ahmed F., Franconi C.J., Munn P.R., Grenier J.K., Hanson M.R. (2024). Transcriptional reprogramming primes CD8+ T cells toward exhaustion in Myalgic encephalomyelitis/chronic fatigue syndrome. Proc. Natl. Acad. Sci. USA.

[161] Chen-Camaño R., DeAntonio R., Lopez-Verges S. (2025). T-cell exhaustion in COVID-19: What do we know?. Front. Immunol..

[162] Jeong J., Choi Y.J., Lee H.K. (2022). The Role of Autophagy in the Function of CD4+ T Cells and the Development of Chronic Inflammatory Diseases. Front. Pharmacol..

[163] Yu Y., Sun B. (2020). Autophagy-mediated regulation of neutrophils and clinical applications. Burns Trauma.

[164] Lam M., Marsters S.A., Ashkenazi A., Walter P. (2020). Misfolded proteins bind and activate death receptor 5 to trigger apoptosis during unresolved endoplasmic reticulum stress. eLife.

[165] Kennedy G., Spence V., Underwood C., Belch J.J.F. (2004). Increased neutrophil apoptosis in chronic fatigue syndrome. J. Clin. Pathol..

[166] Maes M., Twisk F.N., Kubera M., Ringel K., Leunis J.-C., Geffard M. (2012). Increased IgA responses to the LPS of commensal bacteria is associated with inflammation and activation of cell-mediated immunity in chronic fatigue syndrome. J. Affect. Disord..

[167] Pathak A., Agrawal D.K. (2025). Role of gut microbiota in long covid: Impact on immune function and organ system health. Arch. Microbiol. Immunol..

[168] Goldansaz S.A., Hailemariam D., Dervishi E., Zwierzchowski G., Wójcik R., Wishart D.S., Ametaj B.N. (2025). Lipopolysaccharide and recombinant prion protein induce distinct neurodegenerative pathologies in FVB/N mice. Int. J. Mol. Sci..

[169] Jaeger L.B., Dohgu S., Sultana R., Lynch J.L., Owen J.B., Erickson M.A., Shah G.N., Price T.O., Fleegal-Demotta M.A., Butterfield D.A. (2009). Lipopolysaccharide alters the blood-brain barrier transport of amyloid beta protein: A mechanism for inflammation in the progression of Alzheimer’s disease. Brain Behav. Immun..

[170] Wu Y., He Y., Wang F., Yao N., Zhao Y., Tian Z. (2022). Lipopolysaccharide Inhibits Autophagy and Promotes Inflammatory Responses via p38 MAPK-Induced Proteasomal Degradation of Atg13 in Hepatic Stellate Cells. Mediat. Inflamm..

[171] Subedi S., Sasidharan S., Nag N., Saudagar P., Tripathi T. (2022). Amyloid cross-seeding: Mechanism, implication, and inhibition. Molecules.

[172] Kaufman S.K., Diamond M.I. (2013). Prion-like propagation of protein aggregation and related therapeutic strategies. Neurotherapeutics.

[173] Silvestro S., Midiri A., Biondo C., Casilli S., Borrello L., Zummo S., Mancuso G. (2026). Silent Intruders: The Gut Virome in Brain Aging and Cognitive Decline. Pathogens.

[174] Tavassoly O., Safavi F., Tavassoly I. (2020). Seeding brain protein aggregation by SARS-CoV-2 as a possible long-term complication of COVID-19 infection. ACS Chem. Neurosci..

[175] Wang J., Dai L., Deng M., Xiao T., Zhang Z., Zhang Z. (2024). SARS-CoV-2 Spike Protein S1 Domain Accelerates α-Synuclein Phosphorylation and Aggregation in Cellular Models of Synucleinopathy. Mol. Neurobiol..

[176] Duff E.P., Zetterberg H., Heslegrave A., Dehghan A., Elliott P., Allen N., Runz H., Laban R., Veleva E., Whelan C.D. (2025). Plasma proteomic evidence for increased β-amyloid pathology after SARS-CoV-2 infection. Nat. Med..

[177] Basha S., Nadkarni P.P., Pai A.R., Mahato K.K. (2026). Co-aggregation of amyloidogenic proteins in age-related neurodegenerative diseases. Ageing Res. Rev..

[178] Prusiner S.B. (1982). Novel proteinaceous infectious particles cause scrapie. Science.

[179] Rosenberg R.N. (1997). Nobel prize in physiology or medicine for 1997 awarded to Stanley B. Prusiner, MD. Arch. Neurol..

[180] Houston F., Andréoletti O. (2019). Animal prion diseases: The risks to human health. Brain Pathol..

[181] Saitoh Y., Mizusawa H. (2024). Prion diseases, always a threat?. J. Neurol. Sci..

[182] Zhao Y., Jaber V.R., Lukiw W.J. (2022). SARS-CoV-2, long COVID, prion disease and neurodegeneration. Front. Neurosci..

[183] Westman H., Hammarström P., Nyström S. (2025). SARS-CoV-2 spike protein amyloid fibrils impair fibrin formation and fibrinolysis. Biochemistry.

[184] Oh J.-M., Shin H.-Y., Park S.-J., Kim B.-H., Choi J.-K., Choi E.-K., Carp R.I., Kim Y.-S. (2008). The involvement of cellular prion protein in the autophagy pathway in neuronal cells. Mol. Cell. Neurosci..

[185] Thellung S., Scoti B., Corsaro A., Villa V., Nizzari M., Gagliani M.C., Porcile C., Russo C., Pagano A., Tacchetti C. (2018). Pharmacological activation of autophagy favors the clearing of intracellular aggregates of misfolded prion protein peptide to prevent neuronal death. Cell Death Dis..

[186] Meratan A.A., Ghasemi A., Nemat-Gorgani M. (2011). Membrane integrity and amyloid cytotoxicity: A model study involving mitochondria and lysozyme fibrillation products. J. Mol. Biol..

[187] Gonzalez-Garcia M., Fusco G., De Simone A. (2021). Membrane interactions and toxicity by misfolded protein oligomers. Front. Cell Dev. Biol..

[188] Chaudhary H., Iyer A., Subramaniam V., Claessens M.M.A.E. (2016). α-Synuclein oligomers stabilize pre-existing defects in supported bilayers and propagate membrane damage in a fractal-like pattern. Langmuir.

[189] Meratan A.A., Nemat-Gorgani M. (2012). Mitochondrial membrane permeabilization upon interaction with lysozyme fibrillation products: Role of mitochondrial heterogeneity. Biochim. Biophys. Acta (BBA) Biomembr..

[190] Abbasabadi A.O., Javanian A., Nikkhah M., Meratan A.A., Ghiasi P., Nemat-Gorgani M. (2013). Disruption of mitochondrial membrane integrity induced by amyloid aggregates arising from variants of SOD1. Int. J. Biol. Macromol..

[191] Bode D.C., Freeley M., Nield J., Palma M., Viles J.H. (2019). Amyloid-β oligomers have a profound detergent-like effect on lipid membrane bilayers, imaged by atomic force and electron microscopy. J. Biol. Chem..

[192] Fusco G., Chen S.W., Williamson P.T.F., Cascella R., Perni M., Jarvis J.A., Cecchi C., Vendruscolo M., Chiti F., Cremades N. (2017). Structural basis of membrane disruption and cellular toxicity by α-synuclein oligomers. Science.

[193] Zhang H., Jiang X., Ma L., Wei W., Li Z., Chang S., Wen J., Sun J., Li H. (2022). Role of Aβ in Alzheimer’s-related synaptic dysfunction. Front. Cell Dev. Biol..

[194] Jing Y., Kobayashi M., Vu H.T., Kasahara A., Chen X., Pham L.T., Kurayoshi K., Tadokoro Y., Ueno M., Todo T. (2022). Therapeutic advantage of targeting lysosomal membrane integrity supported by lysophagy in malignant glioma. Cancer Sci..

[195] Jiang P., Gan M., Yen S.-H., McLean P.J., Dickson D.W. (2017). Impaired endo-lysosomal membrane integrity accelerates the seeding progression of α-synuclein aggregates. Sci. Rep..

[196] Wang F., Gómez-Sintes R., Boya P. (2018). Lysosomal membrane permeabilization and cell death. Traffic.

[197] Cui Z., Esposito A., Napolitano G., Ballabio A., Hurley J.H. (2025). Structural basis for mTORC1 activation on the lysosomal membrane. Nature.

[198] Resende R., Ferreiro E., Pereira C., Resende de Oliveira C. (2008). Neurotoxic effect of oligomeric and fibrillar species of amyloid-beta peptide 1-42: Involvement of endoplasmic reticulum calcium release in oligomer-induced cell death. Neuroscience.

[199] Soria L.R., Allegri G., Melck D., Pastore N., Annunziata P., Paris D., Polishchuk E., Nusco E., Thöny B., Motta A. (2018). Enhancement of hepatic autophagy increases ureagenesis and protects against hyperammonemia. Proc. Natl. Acad. Sci. USA.

[200] Rodrigo R., Cauli O., Gomez–Pinedo U., Agusti A., Hernandez–Rabaza V., García-Verdugo J.M., Felipo V. (2010). Hyperammonemia induces neuroinflammation that contributes to cognitive impairment in rats with hepatic encephalopathy. Gastroenterology.

[201] Nakatomi Y., Mizuno K., Ishii A., Wada Y., Tanaka M., Tazawa S., Onoe K., Fukuda S., Kawabe J., Takahashi K. (2014). Neuroinflammation in patients with chronic fatigue syndrome/myalgic encephalomyelitis: An 11C-(R)-PK11195 PET study. J. Nucl. Med..

[202] Batool S.T., Ali U., Khan M.I., Mahato R.K. (2026). Neuroinflammation-driven glymphatic dysfunction: Implications for disease progression and drug-based modulation. Ann. Med. Surg..

[203] Rahman M.A., Rahman M.S., Parvez M.A.K., Kim B. (2023). The emerging role of autophagy as a target of environmental pollutants: An update on mechanisms. Toxics.

[204] Martelli F., Hernandes N.H., Zuo Z., Wang J., Wong C.-O., Karagas N.E., Roessner U., Rupasinghe T., Robin C., Venkatachalam K. (2022). Low doses of the organic insecticide spinosad trigger lysosomal defects, elevated ROS, lipid dysregulation, and neurodegeneration in flies. eLife.

[205] Venkatesan R., Park Y.U., Ji E., Yeo E.-J., Kim S.Y. (2017). Malathion increases apoptotic cell death by inducing lysosomal membrane permeabilization in N2a neuroblastoma cells: A model for neurodegeneration in Alzheimer’s disease. Cell Death Discov..

[206] Callahan J.W., Jones C.S., Davidson D.J., Shankaran P. (1983). The active site of lysosomal sphingomyelinase: Evidence for the involvement of hydrophobic and ionic groups. J. Neurosci. Res..

[207] Gao Q. (2019). Oxidative stress and autophagy. Autophagy: Biology and Diseases: Basic Science.

[208] Scherz-Shouval R., Elazar Z. (2007). ROS, mitochondria and the regulation of autophagy. Trends Cell Biol..

[209] Szögi T., Borsos B.N., Masic D., Radics B., Bella Z., Bánfi A., Ördög N., Zsiros C., Kiricsi Á., Pankotai-Bodó G. (2025). Novel biomarkers of mitochondrial dysfunction in Long COVID patients. Geroscience.

[210] Kiffin R., Bandyopadhyay U., Cuervo A.M. (2006). Oxidative stress and autophagy. Antioxid. Redox Signal..

[211] Gouda N.A., Zhakupova A., Abdelaal A.M., Ahmad F., Elkamhawy A. (2025). The interplay involving oxidative stress and autophagy: Mechanisms, implications, and therapeutic opportunities. Exp. Mol. Pathol..

[212] II Timberlake M., Dwivedi Y. (2019). Linking unfolded protein response to inflammation and depression: Potential pathologic and therapeutic implications. Mol. Psychiatry.

[213] Biasizzo M., Kopitar-Jerala N. (2020). Interplay between NLRP3 inflammasome and autophagy. Front. Immunol..

[214] Yun Y., Baek A., Kim D.-E. (2021). Autophagy downregulates NLRP3-dependent inflammatory response of intestinal epithelial cells under nutrient deprivation. BMB Rep..

[215] Gupta S., Cassel S.L., Sutterwala F.S., Dagvadorj J. (2025). Regulation of the NLRP3 inflammasome by autophagy and mitophagy. Immunol. Rev..

[216] Hu C.C., Rodriguez A.J., Chow D.Z., Cardona J.J. (2026). ME/CFS: Phenotypic Overlap with Infection-Associated Syndromes and Craniofacial Sampling for Neuroimmune Investigation. Int. J. Mol. Sci..

[217] Sharif-Askari F.S., Raber J., Alzoubi K.H. (2025). Long COVID and brain inflammation: Unravelling mechanisms and potential therapies. Front. Immunol..

[218] Pradel B., Robert-Hebmann V., Espert L. (2020). Regulation of innate immune responses by autophagy: A goldmine for viruses. Front. Immunol..

[219] Yang Z., Goronzy J.J., Weyand C.M. (2015). Autophagy in autoimmune disease. J. Mol. Med..

[220] Kumar S., Thangakani A.M., Nagarajan R., Singh S.K., Velmurugan D., Gromiha M.M. (2016). Autoimmune responses to soluble aggregates of amyloidogenic proteins involved in neurodegenerative diseases: Overlapping aggregation prone and autoimmunogenic regions. Sci. Rep..

[221] Jia N., Guan H., Zuo Y., Jeong Y.Y., Amireddy N., Rajapaksha G., Gonzalez C.U., Jaber N., Lee Y.-K., Nissenbaum M. (2026). Dysregulation of autophagosome–mitochondria contacts contributes to autophagy dysfunction and neurodegeneration in tauopathy. Proc. Natl. Acad. Sci. USA.

[222] Erickson M.A., Mahankali A.P. (2024). Interactions of serum amyloid A proteins with the blood-brain barrier: Implications for central nervous system disease. Int. J. Mol. Sci..

[223] Rubin L.H., Shi W., Azola A., Bhattacharyya A., Dastgheyb R.M., Wu J., Della Penna C., Parker H., Santiuste I., Ehrenspeck A. (2025). Blood-brain barrier disruption in long COVID and cognitive correlates: A cross-sectional MRI study. Brain Behav. Immun..

[224] Reddy P.H. (2014). Misfolded proteins, mitochondrial dysfunction, and neurodegenerative diseases. Biochim. Biophys. Acta.

[225] Rao K.B.N., Pandey P., Sarkar R., Ghosh A., Mansuri S., Ali M., Majumder P., Kumar K.R., Ray A., Raychaudhuri S. (2022). Stress responses elicited by misfolded proteins targeted to mitochondria. J. Mol. Biol..

[226] Rohrhofer J., Ebner L., Schweighardt J., Stingl M., Untersmayr E. (2025). The clinical relevance of mast cell activation in myalgic encephalomyelitis/chronic fatigue syndrome. Diagnostics.

[227] Weinstock L.B., Brook J.B., Walters A.S., Goris A., Afrin L.B., Molderings G.J. (2021). Mast cell activation symptoms are prevalent in Long-COVID. Int. J. Infect. Dis..

[228] Liu J., Liu S., Zeng L., Tsilioni I. (2023). Amyloid beta peptides lead to mast cell activation in a novel 3D hydrogel model. Int. J. Mol. Sci..

[229] Brambilla M., Fumoso F., Conti M., Becchetti A., Bozzi S., Mencarini T., Agostoni P., Mancini M.E., Cosentino N., Bonomi A. (2025). Low-grade inflammation in long COVID syndrome sustains a persistent platelet activation associated with lung impairment. Basic. Transl. Sci..

[230] Grobbelaar L.M., Venter C., Vlok M., Ngoepe M., Laubscher G.J., Lourens P.J., Steenkamp J., Kell D.B., Pretorius E., Pretorius E. (2021). SARS-CoV-2 spike protein S1 induces fibrin(ogen) resistant to fibrinolysis: Implications for microclot formation in COVID-19. Biosci. Rep..

[231] Biswas U., Roy R., Ghosh S., Chakrabarti G. (2024). The interplay between autophagy and apoptosis: Its implication in lung cancer and therapeutics. Cancer Lett..

[232] Hetz C., Chevet E., Oakes S.A. (2015). Proteostasis control by the unfolded protein response. Nat. Cell Biol..

[233] Ali Y.H., Abbas U., Khalid M.U., Ahmed I., Hussain N., Baloch I., Mahboob H., Zafar A.A., Musawwir U.A. (2026). Integrated immune, apoptotic and mitochondrial gene dysregulation in Long COVID and their association with symptom burden at 10 months post-infection. Sci. Rep..

[234] Vidal A., Barrows B.D., Bao G., Goudzwaard A. (2024). S234 COVID-19 Long COVID and Gut Dysbiosis (Abstract). Am. J. Gastroenterol..

[235] Burger E., Araujo A., López-Yglesias A., Rajala M.W., Geng L., Levine B., Hooper L.V., Burstein E., Yarovinsky F. (2018). Loss of Paneth cell autophagy causes acute susceptibility to Toxoplasma gondii-mediated inflammation. Cell Host Microbe.

[236] Foerster E.G., Mukherjee T., Cabral-Fernandes L., Rocha J.D., Girardin S.E., Philpott D.J. (2022). How autophagy controls the intestinal epithelial barrier. Autophagy.

[237] Lei L., Deng D., Xu W., Yue M., Wu D., Fu K., Shi Z. (2024). Increased intestinal permeability and lipopolysaccharide contribute to swainsonine-induced systemic inflammation. Ecotoxicol. Environ. Saf..

[238] Roura I., Pardo J., Martín-Barceló C., Falcon C., Oltra J., Campabadal A., Bargalló N., Serradell M., Mayà G., Montini A. (2025). Clinical and brain volumetric correlates of decreased DTI-ALPS, suggestive of local glymphatic dysfunction, in iRBD. npj Park. Dis..

[239] Kemp J., Sunnquist M., Jason L.A., Newton J.L. (2019). Autonomic dysfunction in myalgic encephalomyelitis and chronic fatigue syndrome: Comparing self-report and objective measures. Clin. Auton. Res..

[240] Dani M., Dirksen A., Taraborrelli P., Torocastro M., Panagopoulos D., Sutton R., Lim P.B. (2021). Autonomic dysfunction in ‘long COVID’: Rationale, physiology and management strategies. Clin. Med..

[241] Robertson D. (1999). The epidemic of orthostatic tachycardia and orthostatic intolerance. Am. J. Med. Sci..

[242] Su T.-J., Lin C.-H., Liu Y.-L., Hsueh H.-W., Hsieh S.-T., Chao C.-C., Chiang M.-C. (2024). Altered connectivity of central autonomic network: Effects of dysautonomia in hereditary transthyretin amyloidosis with polyneuropathy. Amyloid.

[243] VanElzakker M.B. (2013). Chronic fatigue syndrome from vagus nerve infection: A psychoneuroimmunological hypothesis. Med. Hypotheses.

[244] Zheng Z.S., Simonian N., Wang J., Rosario E.R. (2024). Transcutaneous vagus nerve stimulation improves Long COVID symptoms in a female cohort: A pilot study. Front. Neurol..

[245] Wu T., Zhu W., Duan R., Sun J., Bao S., Chen K., Han B., Chen Y., Lu Y. (2024). Magnetic vagus nerve stimulation ameliorates contrast-induced acute kidney injury by circulating plasma exosomal miR-365-3p. J. Nanobiotechnol..

[246] Luo B., Wu Y., Liu S.-L., Li X.-Y., Zhu H.-R., Zhang L., Zheng F., Liu X.-Y., Guo L.-Y., Wang L. (2020). Vagus nerve stimulation optimized cardiomyocyte phenotype, sarcomere organization and energy metabolism in infarcted heart through FoxO3A-VEGF signaling. Cell Death Dis..

[247] Horowitz M.A., Sussman J.H., Zomalan B., Rendler J., Singh A., Birouty N., Seaton M., Patel S., Gendreau J.L., Abraham M.E. (2026). Vagus nerve stimulation: An update of currently registered clinical trials on ClinicalTrials. Gov. Surg. Neurol. Int..

[248] Ghali A., Lacout C., Ghali M., Gury A., Beucher A.-B., Lozac’h P., Lavigne C., Urbanski G. (2019). Elevated blood lactate in resting conditions correlate with post-exertional malaise severity in patients with Myalgic encephalomyelitis/Chronic fatigue syndrome. Sci. Rep..

[249] Sun W., Jia M., Feng Y., Cheng X. (2023). Lactate is a bridge linking glycolysis and autophagy through lactylation. Autophagy.

[250] Bangen K.J., Clark A.L., Edmonds E.C., Evangelista N.D., Werhane M.L., Thomas K.R., Locano L.E., Tran M., Zlatar Z.Z., Nation D.A. (2017). Cerebral blood flow and amyloid-β interact to affect memory performance in cognitively normal older adults. Front. Aging Neurosci..

[251] Yoshiuchi K., Farkas J., Natelson B.H. (2006). Patients with chronic fatigue syndrome have reduced absolute cortical blood flow. Clin. Physiol. Funct. Imaging.

[252] MacIntosh B.J., Lin S.S., O’HAra F., Churchill N.W., Tam F., Pavel A., Roudaia E., Sekuler A.B., Cheng I., Gao F. (2025). Post COVID-19 condition is associated with altered regional cerebral blood volume as revealed by dynamic susceptibility contrast MRI. Front. Neuroimaging.

[253] Saha A.K., Schmidt B.B.R., Wilhelmy J., Nguyen V., Do J., Suja V.C., Nemat-Gorgani M., Ramasubramanian A.K., Davis R.W. (2018). Erythrocyte deformability as a potential biomarker for chronic fatigue syndrome. Blood.

[254] Saha A.K., Schmidt B.R., Wilhelmy J., Nguyen V., Abugherir A., Do J.K., Nemat-Gorgani M., Davis R.W., Ramasubramanian A.K. (2019). Red blood cell deformability is diminished in patients with Chronic Fatigue Syndrome. Clin. Hemorheol. Microcirc..

[255] Saha A.K., Schmidt B.R., Wilhelmy J., Nguyen V., Do J.K., Suja V.C., Nemat-Gorgani M., Ramasubramanian A.K., Davis R.W. (2019). Altered Erythrocyte Biophysical Properties in Chronic Fatigue Syndrome. Biophys. J..

[256] Guo Y., Zhou S., Ren S., Liu X., Nemat-Gorgani M., Gresser M.J., Davis R.W., Wan J. (2025). Microfluidic assessment of PO2-regulated RBC capillary velocity in ME/CFS. Blood Red. Cells Iron.

[257] Grau M., Presche A., Krüger A.-L., Bloch W., Haiduk B. (2024). Red blood cell morphology is associated with altered hemorheological properties and fatigue in patients with long COVID. Biology.

[258] Voegels R.L., Pinna F.d.R., Imamura R., Farfel J.M., Godoy M.D.C.L. (2015). Olfaction in neurologic and neurodegenerative diseases: A literature review. Int. Arch. Otorhinolaryngol..

[259] De Cleene N., Schwarzová K., Labrecque S., Cerejo C., Djamshidian A., Seppi K., Heim B. (2025). Olfactory dysfunction as potential biomarker in neurodegenerative diseases: A narrative review. Front. Neurosci..

[260] Stigliano E., Tocci A., Florio R., Arena V., Amadoro G. (2026). Olfactory Dysfunction and Cognitive Deterioration in Long COVID: Pathomechanisms and Clinical Implications in Development of Alzheimer’s Disease. Cells.

[261] Lamb M.M., DeHority K., Russel S.M., Kim S., Stack T., Mohammad I., Zeatoun A., Cromwell C.K., Ebert C.S., Baratta J.M. (2023). Characteristics of olfactory dysfunction in patients with long-haul covid-19. Rhinol. Online.

[262] Wostyn P. (2021). COVID-19 and chronic fatigue syndrome: Is the worst yet to come?. Med. Hypotheses.

[263] Sulatsky M.I., Sulatskaya A.I., Stepanenko O.V. (2025). An unexpected insight into the cause of olfactory dysfunction: Fibrillogenesis of odorant-binding proteins. Cell Death Discov..

[264] Finkbeiner S. (2020). The autophagy lysosomal pathway and neurodegeneration. Cold Spring Harb. Perspect. Biol..

[265] Gabig-Cimińska M. (2025). Dysregulated TFEB–autophagy-lysosome pathway links acute COVID-19 immunopathology to Long COVID sequelae. Front. Immunol..

[266] Rabiee A., Ebrahim-Habibi A., Ghasemi A., Nemat-Gorgani M. (2013). How curcumin affords effective protection against amyloid fibrillation in insulin. Food Funct..

[267] Dehghani Z., Meratan A.A., Saboury A.A., Nemat-Gorgani M. (2020). α-Synuclein fibrillation products trigger the release of hexokinase I from mitochondria: Protection by curcumin, and possible role in pathogenesis of Parkinson’s disease. Biochim. Biophys. Acta (BBA) Biomembr..

[268] Shariatizi S., Meratan A.A., Ghasemi A., Nemat-Gorgani M. (2015). Inhibition of amyloid fibrillation and cytotoxicity of lysozyme fibrillation products by polyphenols. Int. J. Biol. Macromol..

[269] Jia Y., Wang N., Liu X. (2017). Resveratrol and amyloid-beta: Mechanistic insights. Nutrients.

[270] Halma M.T., Marik P.E., Saleeby Y.M. (2024). Exploring autophagy in treating SARS-CoV-2 spike protein-related pathology. Endocr. Metab. Sci..

[271] Dossou A.S., Basu A. (2019). The emerging roles of mTORC1 in macromanaging autophagy. Cancers.

[272] Sironi J., Aranda E., Nordstrøm L.U., Schwartz E.L. (2019). Lysosome membrane permeabilization and disruption of the molecular target of rapamycin (mTOR)-lysosome interaction are associated with the inhibition of lung cancer cell proliferation by a chloroquinoline analog. Mol. Pharmacol..

[273] Bar-Tana J. (2025). mTORC1 syndrome (TorS): Unifying paradigm for PASC, ME/CFS and PAIS. J. Transl. Med..

[274] Franco-Juárez B., Coronel-Cruz C., Hernández-Ochoa B., Gómez-Manzo S., Cárdenas-Rodríguez N., Arreguin-Espinosa R., Bandala C., Canseco-Ávila L.M., Ortega-Cuellar D. (2022). TFEB; beyond its role as an autophagy and lysosomes regulator. Cells.

[275] Sardiello M. (2016). Transcription factor EB: From master coordinator of lysosomal pathways to candidate therapeutic target in degenerative storage diseases. Ann. N. Y. Acad. Sci..

[276] Yang J., Zhang W., Zhang S., Iyaswamy A., Sun J., Wang J., Yang C. (2023). Novel insight into functions of transcription factor EB (TFEB) in Alzheimer’s disease and Parkinson’s disease. Aging Dis..

[277] Jia J., Bissa B., Brecht L., Allers L., Choi S.W., Gu Y., Zbinden M., Burge M.R., Timmins G., Hallows K. (2020). AMPK, a regulator of metabolism and autophagy, is activated by lysosomal damage via a novel galectin-directed ubiquitin signal transduction system. Mol. Cell.

[278] Brown A.E., Dibnah B., Fisher E., Newton J.L., Walker M. (2018). Pharmacological activation of AMPK and glucose uptake in cultured human skeletal muscle cells from patients with ME/CFS. Biosci. Rep..

[279] Ashraf M.S., Tuli K., Moiz S., Sharma S.K., Sharma D., Adnan M. (2024). AMP kinase: A promising therapeutic drug target for post-COVID-19 complications. Life Sci..

[280] Sun Q., Fan W., Zhong Q. (2009). Regulation of Beclin 1 in autophagy. Autophagy.

[281] Kang R., Zeh H.J., Lotze M.T., Tang D. (2011). The Beclin 1 network regulates autophagy and apoptosis. Cell Death Differ..

[282] Liu Q., Ye J., Chen C., Zhu Y., He G., Wu F. (2026). Decoding Class III Phosphatidylinositol 3-Kinase (PtdIns3K) Regulation: Mechanistic Insights and Therapeutic Opportunities. ChemMedChem.

[283] Okuyan H.M., Dogan S., Bal T., Çabalak M. (2021). Beclin-1, an autophagy-related protein, is associated with the disease severity of COVID-19. Life Sci..

[284] Liu L., Yan L., Liao N., Wu W.-Q., Shi J.-L. (2020). A review of ULK1-mediated autophagy in drug resistance of cancer. Cancers.

[285] Petherick K.J., Conway O.J., Mpamhanga C., Osborne S.A., Kamal A., Saxty B., Ganley I.G. (2015). Pharmacological inhibition of ULK1 kinase blocks mammalian target of rapamycin (mTOR)-dependent autophagy. J. Biol. Chem..

[286] Hochecker B., Matt K., Scherer M., Meßmer A., von Ardenne A., Bergemann J. (2025). Heat vs. Fatigue: Hyperthermia as a Possible Treatment Option for Myalgic Encephalomyelitis/Chronic Fatigue Syndrome (ME/CFS). Int. J. Mol. Sci..

[287] Pickrell A.M., Youle R.J. (2015). The roles of PINK1, parkin, and mitochondrial fidelity in Parkinson’s disease. Neuron.

[288] Cao F., Li Y., Peng T., Li Y., Yang L., Hu L., Zhang H., Wang J. (2024). PTEN in kidney diseases: A potential therapeutic target in preventing AKI-to-CKD transition. Front. Med..

[289] Miller R., Wentzel A.R., Richards G.A. (2020). COVID-19: NAD^+^ deficiency may predispose the aged, obese and type2 diabetics to mortality through its effect on SIRT1 activity. Med. Hypotheses.

[290] Ruiz-Pablos M., Paiva B., Zabaleta A. (2024). Hypocortisolemic ASIA: A vaccine-and chronic infection-induced syndrome behind the origin of long COVID and myalgic encephalomyelitis. Front. Immunol..

[291] Dodson M., Redmann M., Rajasekaran N.S., Darley-Usmar V., Zhang J. (2015). KEAP1–NRF2 signalling and autophagy in protection against oxidative and reductive proteotoxicity. Biochem. J..

